# Transplantation of Wnt4‐modified neural stem cells mediate M2 polarization to improve inflammatory micro‐environment of spinal cord injury

**DOI:** 10.1111/cpr.13415

**Published:** 2023-02-06

**Authors:** Baiqi Pan, Xiaoyu Wu, Xiaolin Zeng, Jiewen Chen, Wenwu Zhang, Xing Cheng, Yong Wan, Xiang Li

**Affiliations:** ^1^ Department of Joint Surgery, The First Affiliated Hospital Sun Yat‐sen University Guangzhou China; ^2^ Guangdong Province Key Laboratory of Orthopaedics and Traumatology Guangzhou China; ^3^ Department of Spine Surgery, The First Affiliated Hospital Sun Yat‐sen University Guangzhou China

## Abstract

Neural stem cells (NSCs) transplantation has been considered as a potential strategy to reconnect the neural circuit after spinal cord injury (SCI) but the therapeutic effect was still unsatisfied because of the poor inflammatory micro‐environment of SCI. Previous study reported that neuroprotection and inflammatory immunomodulation were considered to be most important mechanism of NSCs transplantation. In addition, Wnt4 has been considered to be neurogenesis and anti‐inflammatory so that it would be an essential assistant agent for NSCs transplantation. Our single cells sequence indicates that macrophages are the most important contributor of inflammatory response after SCI and the interaction between macrophages and astrocytes may be the most crucial to inflammatory microenvironment of SCI. We further report the first piece of evidence to confirm the interaction between Wnt4‐modified NSCs and macrophages using NSCs‐macrophages co‐cultured system. Wnt4‐modified NSCs induce M2 polarization and inhibit M1 polarization of macrophages through suppression of TLR4/NF‐κB signal pathway; furthermore, M2 cells promote neuronal differentiation of NSCs through MAPK/JNK signal pathway. In vivo, transplantation of Wnt4‐modified NSCs improves inflammatory micro‐environment through induce M2 polarization and inhibits M1 polarization of macrophages to promote axonal regeneration and tissue repair. The current study indicated that transplantation of Wnt4‐modified NSCs mediates M2 polarization of macrophages to promote spinal cord injury repair. Our novel findings would provide more insight of SCI and help with identification of novel treatment strategy.

## Graphical abstract

Neural stem cells (NSCs) transplantation is a promising strategy to treat spinal cord injury (SCI). However, the poor inflammatory microenvironment of SCI is still the barrier for it to reach satisfied therapeutic effect in clinic. Here, we unveiled that transplantation of Wnt4‐modified NSCs effectively improves the inflammatory microenvironment through inducing M2 polarization and suppressing M1 polarization of macrophages after SCI. Wnt4 may be a remarkable potential therapeutic target in NSCs transplantation for SCI.

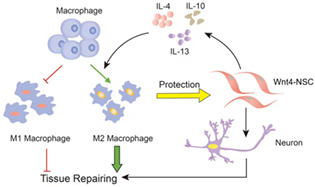

## 1 INTRODUCTION

Traumatic injury of the spinal cord disrupts the descending and ascending axonal tracts and interrupts the communication between the brain and body, leading to the loss of motor, sensory and autonomic functions.^1^, ^2^, ^3^ Although, the incidence of spinal cord injury (SCI) is relatively low, it often results in disastrous emotional, social and economic impacts on the injured person and their family, community, even society.^3^ To date, there are still no sufficient therapies for SCI and the only therapeutic option for SCI patients is physical rehabilitation.^4^, ^5^ Reconstruction of the damaged neural circuits was once considered to be hopeless as the adult mammalian central nervous system has very poor ability to regenerate.^2^, ^6^ Cell transplantation has been considered as a potential strategy to reconnect the neural circuit after SCI.^1^, ^2^, ^7^ Several studies have reported that neural stem cells (NSCs) transplantation results in partial repairing due to the neuronal differentiation of NSCs.^7^ However, several studies reported that most exogenous NSCs at the lesion site differentiated into astrocytes, which are to the disadvantage of spinal cord repairing, rather than Neuron.^6^, ^8^ Thus, the outcome of NSCs transplantation was not satisfied.

Following transplantation of NSCs, the transplanted cells were considered to mediate functional improvements following SCI through a variety of mechanisms, including axonal regeneration, neuroprotection and inflammatory immunomodulation.^2^ Many repair mechanisms rely on beneficial aspects of inflammation. However, the excessive inflammation led to lots of cell death and generation of astrocyte which are harmful to tissue repair and axonal regeneration^9^, ^10^, ^11^, ^12^, ^13^ Macrophages/microglia lineages play crucial roles in the pathophysiology of many repair including SCI.^14^, ^15^ Macrophages/microglia after injury are organized on a continuum of phenotypes and these phenotypes have been defined as two major categories of proinflammatory M1 cells and anti‐inflammatory M2 cells according to their primary functions.^16^, ^17^ Both these cells represent the extreme activation states of monocyte and macrophages lineages.^17^ M1 cells initiate the inflammation by releasing proinflammatory cytokines, nitric oxide and reactive oxygen species that lead to glial scar formation, neuronal cell apoptosis and causing activation of astrocytes. M2 response to promote tissue repair by secreting anti‐inflammatory cytokines to enhance axonal growth and promote the proliferation and differentiation of oligodendrocyte progenitor cells.^17^, ^18^, ^19^ In fact, both M1 and M2 are important in the whole inflammatory response respectively. However, in SCI, it has been demonstrated that the lesion site of spinal cord is continuously filled with M1 cells and these cells cannot switch to M2 cells that leading to irreversible tissue impairing.^19^ Thus, it is important to elevate the ratio of M2 in the micro‐environment to promote tissue repair in SCI treatment. Previous studies reported that cell transplantation can improve the immune response by increasing anti‐inflammatory cytokines and reducing proinflammatory cytokines after SCI.^14^ However, the involving mechanism is still unclear. Still, few studies investigated the direct interactions between transplanted cells and immune cells; instead, they measure global changes in cytokine release or the abundance of immune cells after SCI.^20^, ^21^

Recently, lot of wingless‐type MMTV integration site family (Wnt) proteins such as Wnt4, Wnt5a, Wnt7b, has gain growing attention as an attractive factor for neural differentiation and anti‐inflammation.^22^ Wnt4 has been considered to have dual role of neuronal differentiation and anti‐inflammation and has been proven to be an optimal assistant agent in cell transplantation after SCI.^23^, ^24^ Wnt4‐modified NSCs have been considered to promote neuronal differentiation and functional recovery in SCI model according to our previous studies.^24^ However, the effect and relative mechanism of Wnt4‐modified NSCs on monocyte/macrophages niche and immune response in inflammatory micro‐environment of SCI are still elusive.

In the current study, we investigated the effect and underlying mechanism of Wnt4‐modified NSCs on macrophages in inflammatory micro‐environment, the potential application of Wnt4‐modified NSCs transplantation in SCI, that may provide useful information for translational application of NSCs transplantation.

## 2 MATERIALS AND METHODS

### 2.1 scRNA‐seq dataset

scRNA‐seq data was downloaded from the GEO database (GSE162610) and clustered by the Seurat package (version 4.0.2).^25^ Highly variable genes were calculated using Seurat ‘FindVariableGenes’ function. Then we performed principal component analysis (PCA) using HVGs and significant top 20 principal components (PCs) were selected to perform UMAP dimensionality reduction. We choose ‘Harmony’ to remove batch effects. UMAP was used to visualize the single cells (a total of 66,178 cells). Unbiased clustering generated 16 main clusters and was annotated to 10 known cell types according to canonical marker genes. Differential expression genes (DEGs, adjusted *p* < 0.05, |log2 fold change| >0.5) were identified by ‘FindMarkers’ function in the Seurat package. Gene Set Variation Analysis (GSEA) was conducted using ClusterProfiler (version 4.0.0) and GSVA package. The gene sets of KEGG pathways were obtained from MSigDB (https://www.gsea-msigdb.org/gsea/index.jsp). Pseudotime Analysis was conducted using Monocle (version 2.18.0) package. Cell–cell interactions were inferred by CellChat (version 1.13) package.

### 2.2 NSCs isolation and culturing

NSCs cultures were obtained from the foetal brains of embryonic day 14 rats, which were extracted from pregnant Sprague–Dawley (SD) rats (Laboratory Animal Center of Sun Yat‐sen University, Guangzhou, China) and identified as previously described.^24^, ^26^ Briefly, the brain tissue was mechanically dissected and dissociated in Hanks Balanced Salt Solution to prepare cell suspensions which were centrifuged at 1000 rpm for 5 min. The cell pellet was diluted to a single‐cell suspension after the supernatant was discarded. NSCs were plated on a T25 culture flask (Corning, Acton, MA) containing Dulbecco modified eagle medium (DMEM)/F‐12 nutrient mixture, 2% B27, 1% penicillin/streptomycin, 1% l‐glutamine (Gibco, Grand Island, NY), 20 ng/mL fibroblast growth factor‐2 (FGF‐2) and 20 ng/mL epidermal growth factor (EGF) (Peprotech, Rocky Hill, NJ). NSCs were cultured at 37°C in 5% CO_2_ and were passaged via weekly digestion with accutase (Millipore, Bedford, MA) in the medium described above. All NSCs used in this research were between passages 2 and 4.

To induce neural differentiation, cells were plated at a density of 2 × 10^5^ cells/well in 6‐ or 12‐well tissue‐culture plates and allowed to adhere for 24 h at 37°C, at which time cells were switched to neural differentiated medium consisting of basic medium supplemented with 2% B27, 1% penicillin/streptomycin, 1% l‐glutamine. The medium was changed every 2–3 days.

Monocytes were isolated from the femurs of adult SD rats (Laboratory Animal Center of Sun Yat‐sen University, Guangzhou, China) as previously described.^27^ To induce M1 and M2 polarization of macrophages, cells were plated at a density of 2 × 10^5^ cells/well in 6‐ or 12‐well tissue‐culture plates and allowed to adhere for 24 h at 37°C, at which time cells were switched to polarization medium consisting of basic medium supplemented with LPS (10 μg/mL) for M1 and IL‐4 (10 ng/mL) for M2 polarization (Figure S1A–C).^28^, ^29^

### 2.3 Lentiviral vector construction

The lentiviral vectors carrying green fluorescent protein (GFP) and a sequence that specifically overexpression the Wnt4. Lentiviruses carrying GFP and Wnt4 gene overexpression were constructed as previously described^24^ (Figure S2A–C). Briefly, the full‐length Rat Wnt4 gene was encoded into vectors. The vectors and corresponding packaging plasmids were co‐transfected into 293T cells using lipofectamine 2000 (Invitrogen, Camarillo, CA). The medium was changed to complete medium after 8 hours‐incubation. After 48 h, the supernatant was harvested from 293T cells, filtered using a 0.45‐mm pore size filter, and concentrated via ultracentrifugation at 96,500 g for 2 h at 4°C. The serially diluted lentivirus was used to transduce 293T cells after resuspension. Then, the labelled 293T cells were counted to calculate the viral titre, and high‐titre recombinant lentiviral vectors carrying Wnt4 were harvested after 4 days.

### 2.4 Surgical procedures and cell transplantation

Adult female Sprague–Dawley (SD) rats (weighing 200–220 g, supplied by the Experimental Animal Center of Sun Yat‐sen University, Guangzhou, China) were divided into four groups for this study: Sham group (*n* = 10), SCI group (*n* = 10), NSC^vector^ group (*n* = 10), and NSC^Wnt4^ group (*n* = 10). After 72 h of lentiviral transfection, NSCs were collected for transplantation. Briefly, animals were anaesthetized with 1% pentobarbital sodium (40–45 mg/kg), and the spinal cord was exposed at the 10th thoracic vertebral level (T10) via laminectomy. The animals underwent spinal cord exposure with no injury in the sham group. In the other three groups, the exposed spinal cord was contused with a weight‐drop device by dropping a 10 g rod from a precalibrated height of 12.5 mm.^30^ Once the bleeding had stopped, 5 μL NSCs were implanted at a density of 1 × 10^5^ cells/μL to the rostral and caudal of injured site using microsyringe.^31^, ^32^ After SCI and NSCs transplantation, the T8‐T11 spinal cord segments were dissected at 8 weeks.

### 2.5 Hindlimb locomotor scale

The 22‐point (0–21) Basso, Beattie, and Bresnahan (BBB) open‐field locomotor scale was used to assess hindlimb locomotor function, including joint movements, stepping ability, coordination, and trunk stability. A score of 21 indicates unimpaired locomotion as observed in uninjured rats. All animals underwent behavioural testing, and the duration of each session was 5 min per rat. Finally, we calculated the overall score. The evaluation was performed by three independent observers who were blinded to the treatment group of the tested animals.^33^

### 2.6 Footprint analysis

Hindlimb and locomotor behaviour were assessed at 8 weeks post‐injury. The fore and hind limbs were coated with different colours dyes, and the rats of different groups were placed on a 10 cm × 100 cm runway which was covered by a paper. The rats were encouraged to run in a straight line in order to obtain and evaluate the gait of the rats in the different groups. Then the digital footprints were shown as representative pictures to assess the coordination variability.^34^

### 2.7 Spinal cord–evoked potential (SCEP) recording

At 8 weeks post‐injury, total 20 rats (*n* = 5 in the sham group and *n* = 5 in each cell‐transplanted group) were anaesthetized with 1% pentobarbital sodium (40–45 mg/kg) and fixed stereotaxically. The T5‐L1 vertebrae were exposed completely. Briefly, the stimulation electrode was inserted into the T5‐T6 interspinous ligaments, and a pair of needle electrodes was inserted into the interspinous ligaments of T12‐L1 for SCEP recording. Then, the electrodes were connected to a BL‐420 Biological Function Experiment System (Taimeng, Chengdu, China). The variables of the SCEP signals were set according to previous reports as follows: gain of 2000 time constant of 0.01 s, and filtering at 300 Hz. A single pulse stimulation (50 ms in duration at a frequency of 5.1 Hz and a voltage increase of 1 mV) was transmitted to elicit a SCEP through the electrodes until a mild twitch of the vertebral body of the animal was observed. One hundred SCEP responses were averaged for each rat to obtain high‐quality waveforms for the SCEP signals.^35^

### 2.8 Histological analysis

At 8 weeks after SCI, all rats were deeply anaesthetized with an adequate dose of 10% chloral hydrate (5 mL/kg) and transcranial perfusion was used with 250 mL of 0.9% normal saline. Animals were perfused with 300 mL of 4% paraformaldehyde (PFA) in 0.1 M phosphate buffer solution (PBS; pH 7.4). The T8‐T11 cord segments were dissected based on the dorsal spinal root count, post fixed in 4% PFA overnight, and soaked in 10% sucrose followed by 30% sucrose at 4°C overnight. Samples were embedded in optimal cutting temperature compound, frozen at −20°C and sliced in the longitudinal or transverse plane at a thickness of 20 μm.

Animals (*n* = 5 per group) were killed for haematoxylin‐eosin (HE) staining to visualize the cavity area. The T8‐T11 longitudinal spinal cord sections from each group were stained with HE according to standard protocols and examined under bright field microscope.^24^, ^35^

For neuron counting, animals (*n* = 5 per group) were killed, and transverse sections of the injured spinal cord were used to stain neurons with Nissl. Sections at 2 mm rostral and caudal to the lesion epicentre were counted for each rat. The numbers of positively stained cells were counted and averaged per section in a blinded manner.^24^, ^35^

For axonal tract tracing, dorsal laminectomy was performed at T12 and Fluorogold (FG; Biotium, Fremont, CA) was injected into the spinal cord at 7 weeks after operation. One week after injection, the animals were perfused and T8 segment of the spinal cord was removed, cryopreserved, embedded in OCT compound, and sliced into 10 μm frozen sections. A fluorescence microscope (Olympus, Tokyo, Japan) was used to detect FG‐labelled neurons.^31^, ^32^

### 2.9 Statistical analysis

Data obtained from experiments in triplicate and repeated at least three times were represented as mean ± SD. Statistical evaluations were analysed via one‐way analysis of variance (ANOVA) with Levene's homogeneity of variance test and, subsequently, Bonferroni's post hoc test or Dunnett's T3 post hoc test based on the comparison to be made and the statistical indication of each test. The analyses were performed using Statistical Package for Social Sciences, Version 16.0 for Windows (SPSS, Chicago, IL, USA). A statistical probability of *p* < 0.05 was considered significant.

## 3 RESULTS

### 3.1 Molecular identification of immune and glial cells at the injured site of spinal cord

**FIGURE 1:**
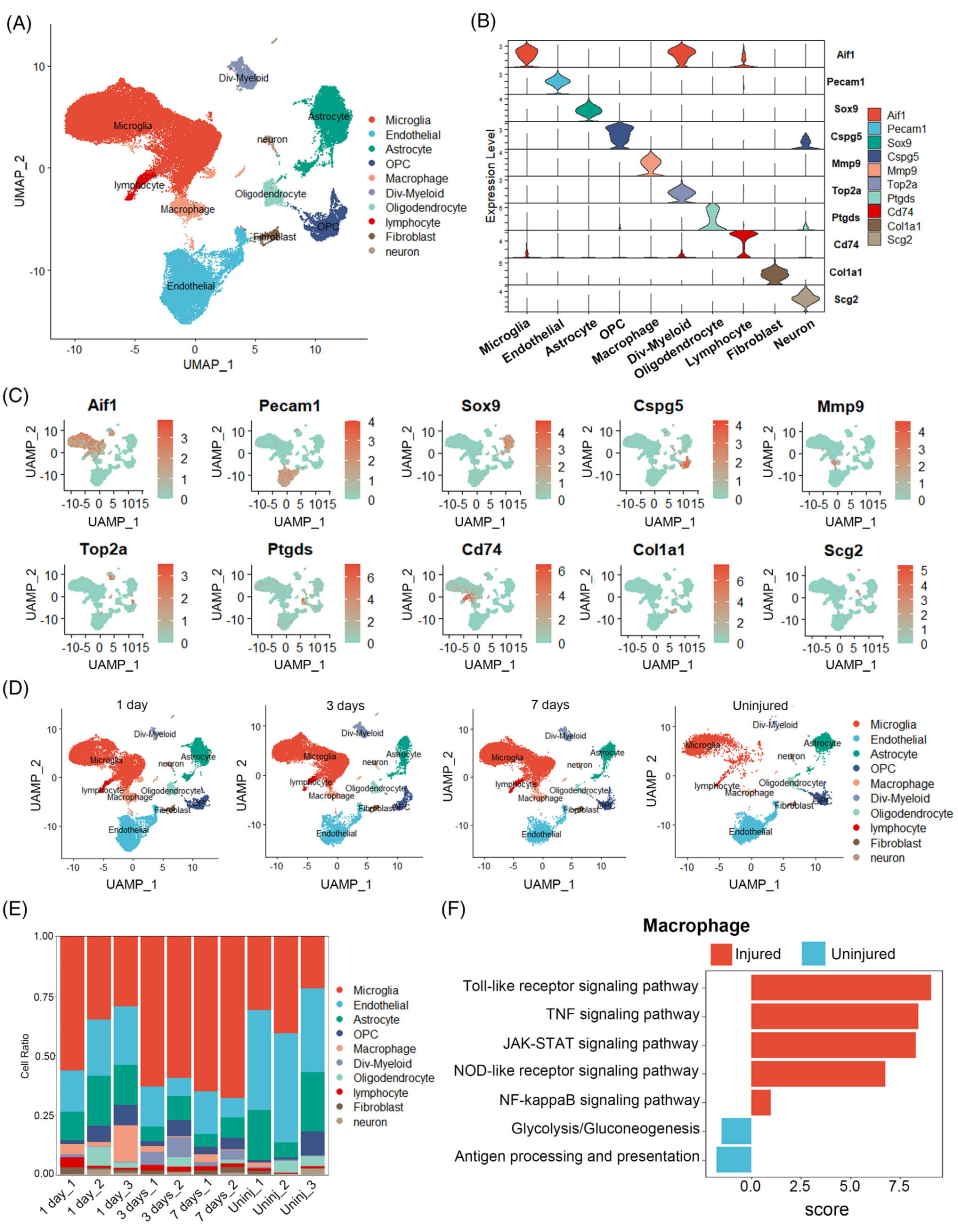
Cellular atlas of mouse spinal cord. (A) UMAP plots showing cell types for the 66,178 cells. (B) Violin plots showing the smoothed expression distribution of marker genes in nine cell types. (C) UMAP plots showing the expression levels of marker genes for 10 cell types. (D) UMAP plots of each time point shows temporal progression of each major cell type. (E) The proportion of each cell type in 10 samples. (F) Gene Set Variation Analysis (GSVA) between injury and control group in macrophages showing the activation of inflammatory pathways.

To assess the cellular heterogeneity among all cell populations at the injury site, we obtained a total of 66,178 cells from uninjured and 1, 3 and 7 dpi tissue, which resulted in a total of 10 distinct clusters when visualized on a UMAP plot (Figure 1A,D). These 10 clusters represented all major cell types that are known to comprise the SCI site including Microglia, Endothelial cells, Astrocytes, OPC, Macrophages, Div‐Myeloid cells, Oligodendrocytes, Lymphocytes, Fibroblasts and Neurons. The highest differentially expressed genes (DEGs) provided a unique molecular signature for each cell type, which in most cases were different from canonical markers used in the previous studies (Figure 1B,C). The proportion of each cell lineage varies greatly among different samples (Figure 1E). Gene Set Variation Analysis (GSVA) between injury and control group in macrophages indicated the activation of inflammatory pathways was observed in macrophage (Figure 1F). Our results presented a single‐cell transcriptome atlas of Mus musculus.

### 3.2 Macrophages but not microglia were the major contributor in inflammatory response of SCI

**FIGURE 2:**
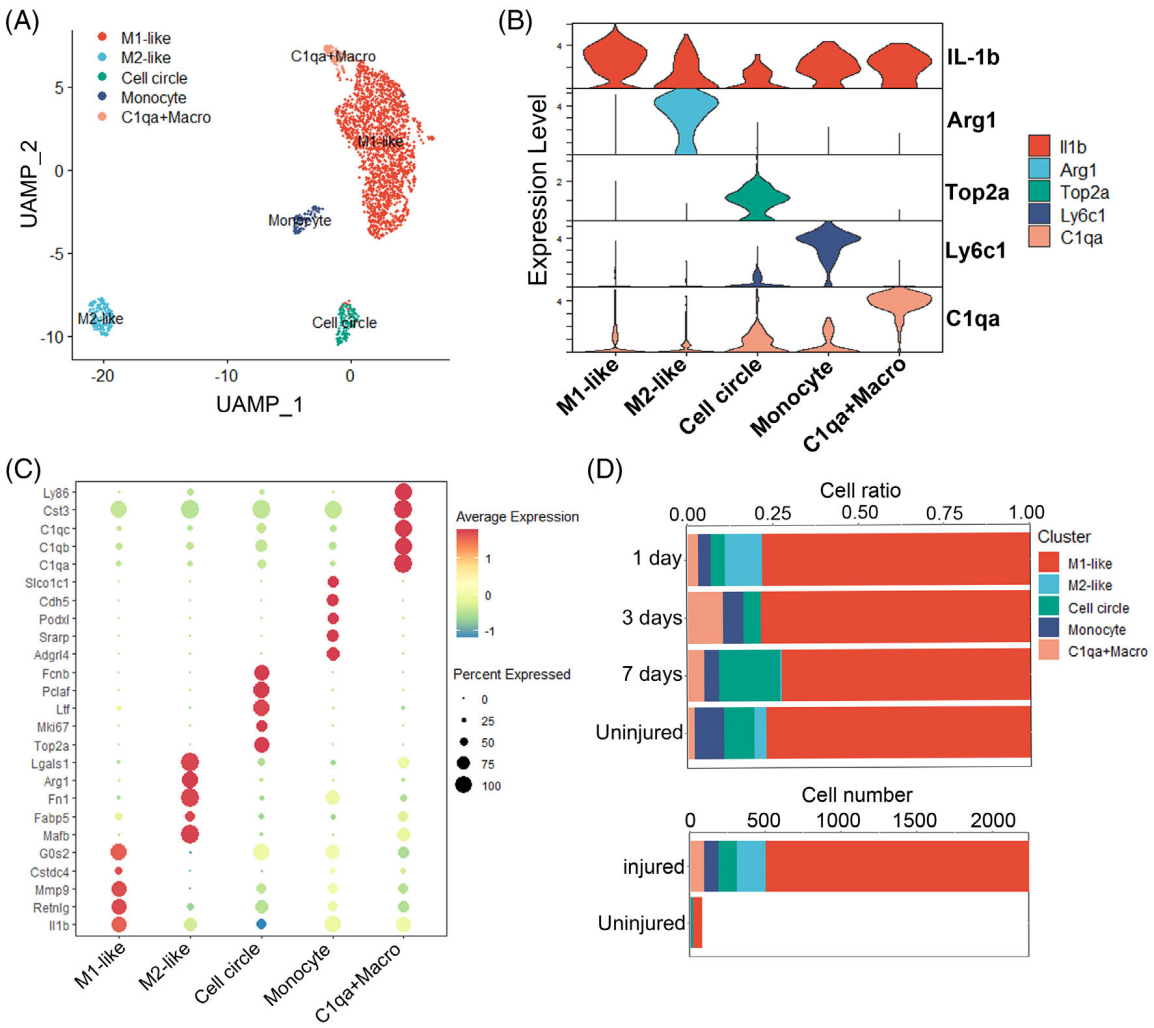
Molecular profile of macrophages subtype heterogeneity acutely after SCI. (A) UMAP plots of macrophages subsets. (B) Violin plots showing the smoothed expression of marker genes in five macrophages subsets. (C) Dot plots showing the high expression of marker genes in five macrophages subsets. (D) The proportion and number of each cell type of macrophages in different groups.

Microglia are the main cell type in SCI. However, the scRNA‐seq results indicated that activated microglia were increased after SCI, these cells may not involve in the inflammatory response (detailed results were in Figure S3). Thus, we focused on macrophages subtypes and its function in SCI. Clustering analysis was performed on macrophages and visualized on a separate UMAP (Figure 2A). Macrophages were divided into five subtypes and M1‐like macrophages were identified by expression of IL1‐b, whereas M2‐like macrophages were identified by Arg1 (Figure 2B,C). Although the proportion of M1‐like macrophages was not changed significantly among uninjured and 1, 3 and 7 dpi group, the numbers of M1‐like macrophages were significantly increased (Figure 2D). The activation of inflammatory pathways was increased in macrophages after SCI. Taken together, our analysis identified M1‐like macrophages were increased in the injury group, indicating that macrophages were the major contributor in the inflammatory environment of SCI.

### 3.3 Macrophages were as major contributor to have various interaction with astrocytes

Previous studies demonstrated that the activation of astrocytes was associated with inflammatory response modulated by M1‐like macrophages.^12^ Ascc1, Nes, Bcan were used to identify naive, reactive and scar‐forming astrocytes subtypes according to previous study.^36^ The scRNA‐seq results (detailed results were in Supporting Information) showed the differentiation paths from naive to scar‐forming astrocytes and indicated naive astrocytes would be differentiated into scar‐forming astrocytes after SCI (Figure S4). This analysis indicated that astrocytes become activated in SCI which contributes to scar formation and is to the disadvantage of tissue repair.

**FIGURE 3:**
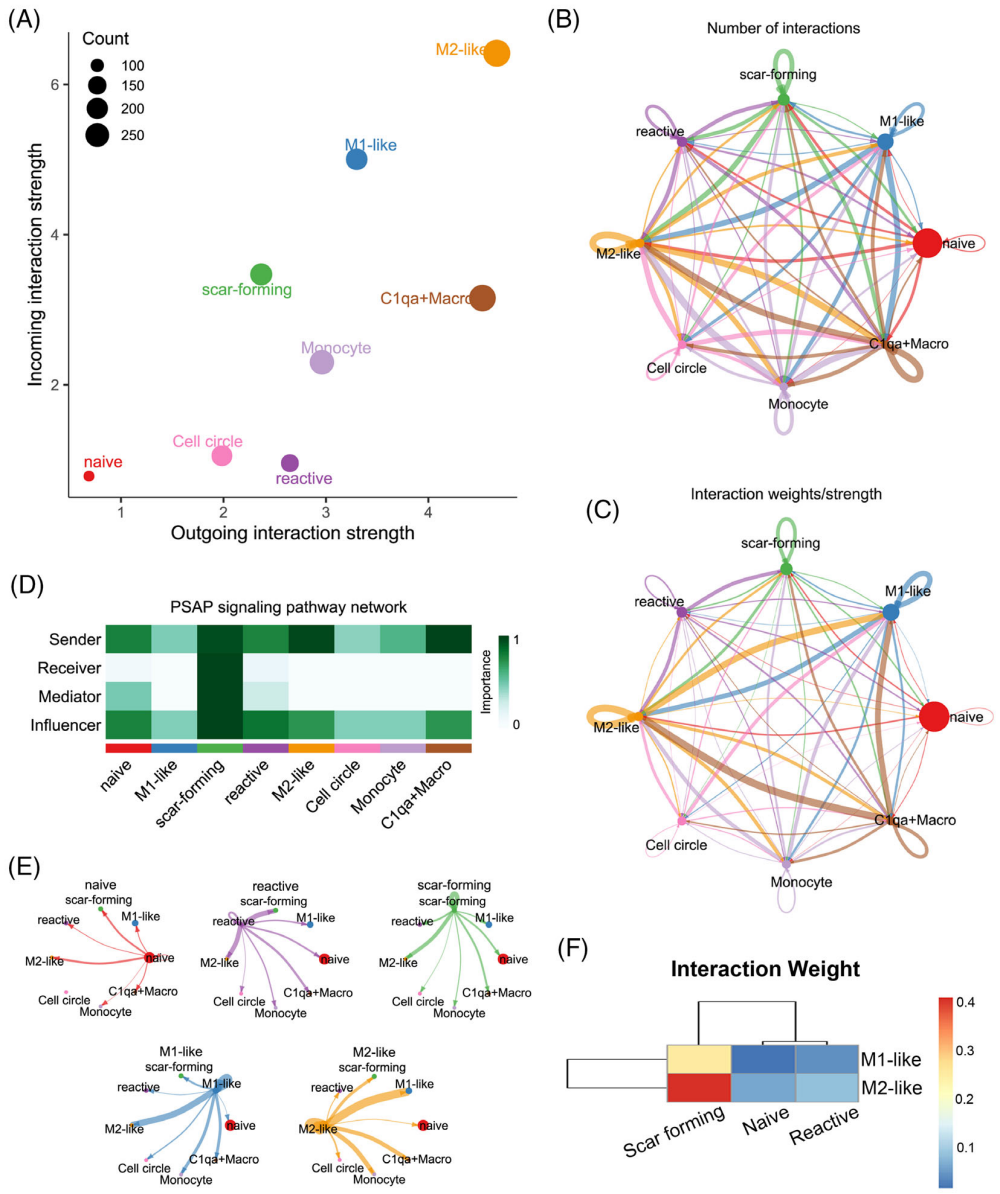
Interaction analysis between macrophages and astrocytes subsets. (A) Scatter plots of signalling role analysis on the aggregated cell–cell communication network from all signalling pathways. (B–C) Circle plot summarizing the maximum number of interactions and the interaction weights/strength among macrophages and astrocytes subsets. (D) PSAP signal pathway network among macrophages and astrocytes subsets. (E) Signals sent by each cell subset. (F) Heatmap summarizing the interaction weights among macrophages and astrocytes subsets.

We further investigated the interaction between macrophages and astrocytes. We compared the outgoing and incoming interaction strength in 2D space allows ready identification of the macrophages and astrocytes subtypes with significant changes in receiving signals. M1 and M2‐like macrophages play an important role in sending signals (Figure 3A). There were various communications between macrophages and astrocytes subtypes (Figure 3B,C,E). Particularly, M1 and M2 macrophages had the strong interaction with scar forming astrocytes (Figure 3D,F). These results confirmed the obvious interaction between macrophages and astrocytes subtypes.

In summary, the single cell analysis indicated that macrophage was the most important contributor in the inflammatory micro‐environment of SCI. The increasing M1‐like macrophages induced the irreversible inflammation to lead to most of cell death; Moreover, the interaction between M1 and astrocytes may be most crucial obstacle to tissue repair in the inflammatory micro‐environment of SCI.

### 3.4 Wnt4‐modified NSCs promote M2 polarization of macrophages through suppression of TLR/NF‐κB signal pathway

**FIGURE 4:**
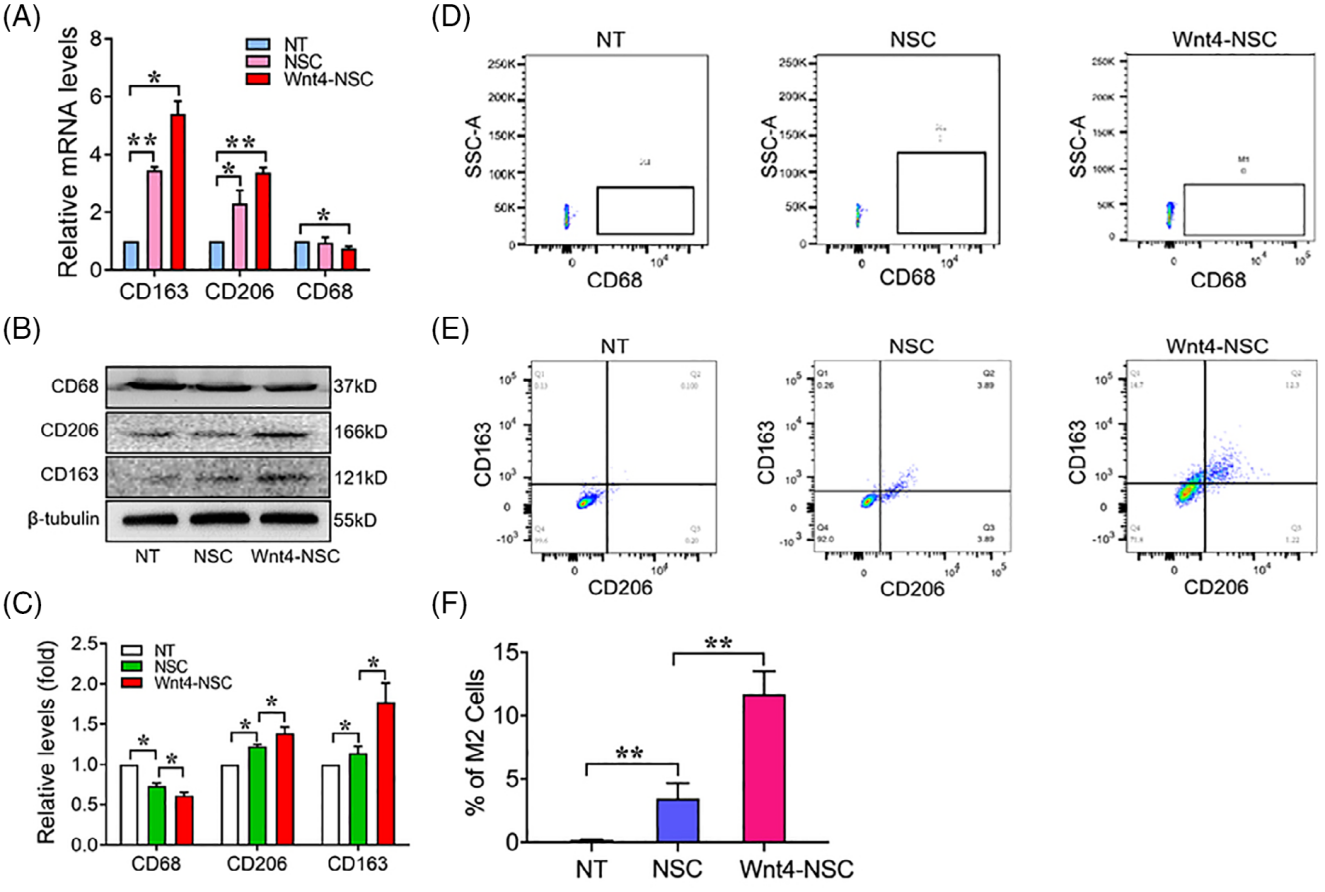
Wnt4‐modified NSCs promote M2 polarization of macrophages. (A) RT‐qPCR analysis of expressions of CD163, CD206 and CD68 in macrophages co‐cultured with NSCs, *n* = 3. (B) Western Blot analysis of expressions of CD163, CD206 and CD68 in macrophages co‐cultured with NSCs. (C) Quantification of western blot data in panel B, *n* = 3. (D) Flow cytometric analyses of M1 cells (CD68^+^) in macrophages co‐cultured with NSCs, *n* = 3. (E) Flow cytometric analyses of M2 cells (CD163^+^ CD206^+^) in macrophages co‐cultured with NSCs, *n* = 3. (F) Quantification of FCAS data in panel E, *n* = 3. (The data are presented as the means ± SD from one representative experiment of three independent experiments performed in triplicate. **p < 0.01 compared between groups; *p < 0.05 compared between groups.)

We first investigated the effect of Wnt4 on NSCs and found that Wnt4 could promote NSCs to generate multiple anti‐inflammatory cytokines including IL‐4, IL‐10, IL‐13, IFN‐γ and NT‐3 (detailed results were in Figure S5). According to our scRNA‐seq results, M1‐like macrophages maybe the most important inflammatory contributor after SCI. Thus, we further investigated the interaction of NSCs and macrophages. Macrophages were co‐cultured with wild type NSCs or Wnt4‐modified NSCs to establish the co‐culture system (Figure S6B). PCR and WB results showed that the M2 relative makers including CD163 and CD206 were increased in macrophages co‐cultured with wild type NSCs and further increased in macrophages co‐cultured with Wnt4‐modified NSCs at mRNA and protein levels. In contrast, M1 makers CD68 were decreased in macrophages co‐cultured with wild type NSCs or Wnt4‐modified NSCs at protein levels (Figure 4A–C). The results of FACS demonstrated that macrophages tended to polarize into M2 cells rather than M1 cells when co‐cultured with Wnt4‐modified NSCs (Figure 4D–F). These results suggested that Wnt4‐modified NSCs promote M2 polarization of macrophages.

**FIGURE 5:**
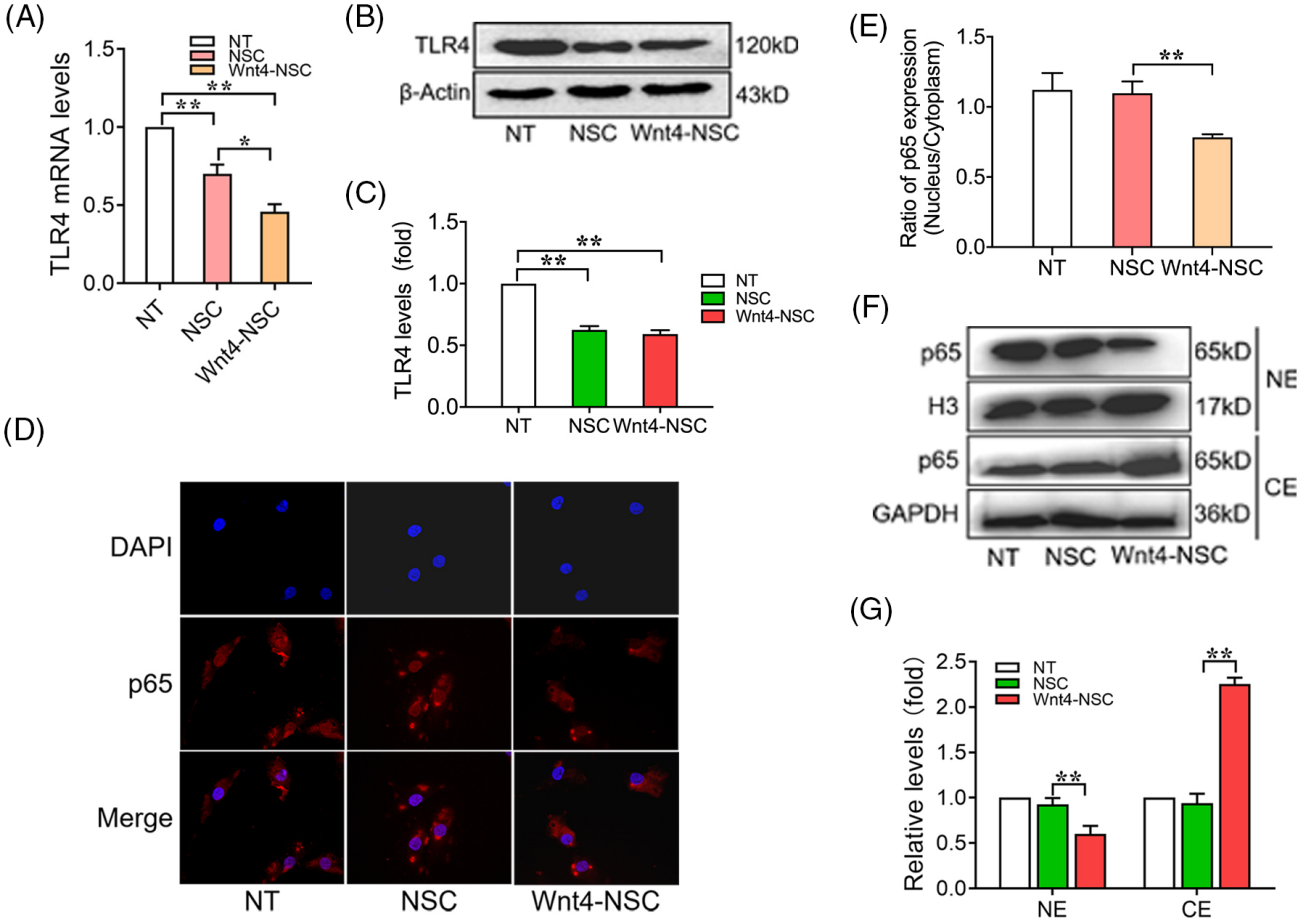
Wnt4‐modified NSCs promote M2 polarization through suppressing of TLR4/NF‐κB signal pathway. (A) RT‐qPCR analysis of expressions of TLR4 in macrophages co‐cultured with NSCs, *n* = 3. (B) Western Blot analysis of expression of TLR4 in macrophages co‐cultured with NSCs, *n* = 3. (C) Quantification of western blot data in panel B, *n* = 3. (D) Immunofluorescence analysis of expression of p65 in macrophages co‐cultured with NSCs, *n* = 3, bar: 10 μm. (E) Quantification of immunofluorescence data in panel D, *n* = 3. (F) Western blot analysis of expression of p65 in nuclear extract (NE) and cytosolic extract (CE) of macrophages co‐cultured with NSCs. (G) Quantification of western blot data in panel E, *n* = 3. (The data are presented as the means ± SD from one representative experiment of three independent experiments performed in triplicate. **p < 0.01 compared between groups; *p < 0.05 compared between groups.)

Toll like receptor 4 (TLR4)/nuclear factor‐κB (NF‐κB) signalling was considered to a critical signalling pathway in neuroinflammation^37^, ^38^ and M1 polarization of macrophages.^38^ We further confirm whether the TLR4/NF‐κB signalling was involved in anti‐inflammatory effect of Wnt4‐modified NSCs. We first examined the mRNA and protein levels of TLR4. The results showed that the expression of TLR4 was significantly decreased in macrophages co‐cultured with wild type NSCs and further decreased in macrophages co‐cultured with Wnt4‐modified NSCs (Figure 5A–C). The immunofluorescence staining showed that the expression and nucleus translocation of p65 (NF‐κB) was significantly decreased in macrophages co‐cultured with Wnt4‐modified NSCs (Figure 5D,E). Immunoblots of p65 in cytosolic extract (CE) and nuclear extract (NE) revealed that p65 nuclear translocation was significantly suppressed in macrophages co‐culture with Wnt4‐modified NSCs (Figure 5F,G). These results suggested that Wnt4‐modified NSCs promote M2 polarization through suppressing of TLR4/NF‐κB signal pathway.

### 3.5 M2 cells promote NSCs differentiate into Neuron through MAPK/JNK signal pathway

The appropriated micro‐environment can elevate the neuronal differentiation rate of transplanted NSCs which is the key point to therapeutic effect of NSCs transplantation. Macrophages are crucial for the inflammatory response in the spinal cord.^39^, ^40^ The appropriate activation of macrophages can aid in the axonal regeneration and tissue repair. Thus, we next investigated the effect of macrophages on neuronal differentiation of NSCs. The immunofluorescence results showed that β3‐tubulin and MAP2 positive cells were significantly decreased in NSCs co‐cultured with M1 cells and increased in NSCs co‐cultured with M2 cells (Figure S7A,B,D,E). In contrast, GFAP positive cells were significantly increased in NSCs co‐cultured with M1 cells and decreased in NSCs co‐cultured with M2 cells (Figure S7C–E). Similar results of RT‐qPCR and WB analyses showed that neurogenic markers expression including β3‐tubulin, MAP2 and GFAP in mRNA and protein levels (Figure S7F–H). We further investigated the involved mechanism of promoting neuronal differentiation of NSCs by M2 cells. The results showed that the effect of M2 cells on promoting neuronal differentiation was abolished in NSCs with JNK specific inhibitor (SP600125) stimulation (detailed results were in Figure S8). These results suggested that M2 cells promote neuronal differentiation through activation of MAPK/JNK signal pathway.

### 3.6 Transplantation of Wnt4‐modified NSCs improved inflammatory micro‐environment to promote axonal regeneration and suppress tissue destruction in SCI

**FIGURE 6:**
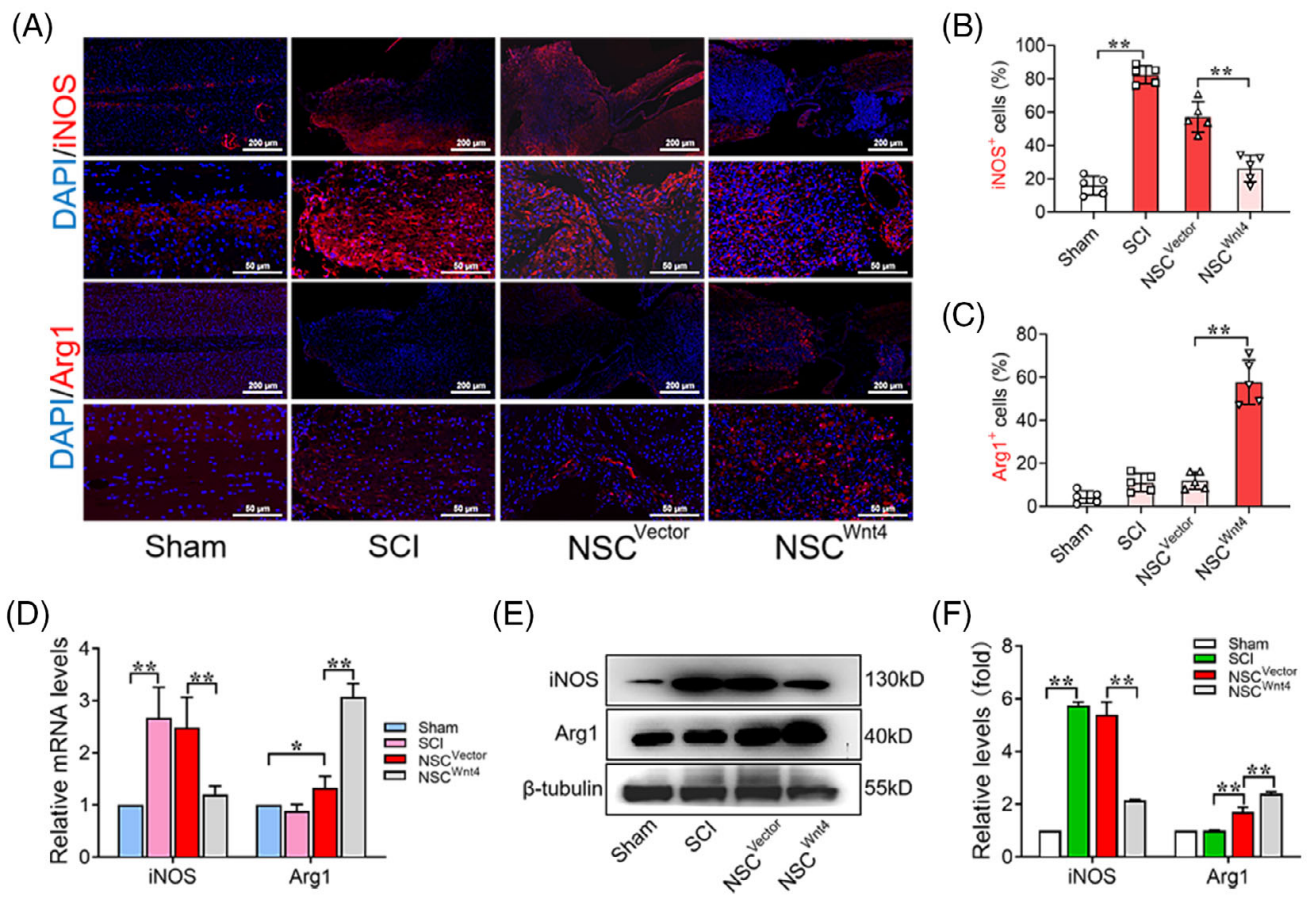
Transplantation of Wnt4‐modified NSCs mediates M2 polarization of macrophages in the inflammatory micro‐environment of SCI. (A) Immunofluorescence analysis of iNOS^+^ and Arg1^+^ cells (red) at the injured site of spinal cord in different groups, bar: 500 μm in upper row; 100 μm in lower row. (B, C) Quantification of immunofluorescence data in panel A, *n* = 5. (D) RT‐qPCR analysis of expressions of iNOS and Arg1 in the spinal cord tissue in the different groups, *n* = 3. (E) Western Blot analysis of expressions of iNOS and Arg1 in the spinal cord tissue in the different groups, *n* = 3. (F) Quantification of western blot data in panel E, *n* = 3. (The data are presented as the means ± SD. **p < 0.01 compared between groups; *p < 0.05 compared between groups.)

As mentioned above, the polarization of macrophages in inflammatory micro‐environment is related to the therapeutic effect of NSCs transplantation because of inducing apoptosis and death of drafted cells and neuron by M1 cells.^41^ Whether transplantation of Wnt4‐modified NSCs mediates the polarization of macrophages is still elusive. Thus, we further investigated the effect of Wnt4‐modified NSCs on polarization of macrophages in the inflammatory micro‐environment of SCI. The immunofluorescence results showed that increasing M1 cells (iNOS^+^) were accumulated at the injured site of spinal cord. Interestingly, The M1 cells were significantly decreased and M2 cells (Arg1^+^) were significantly increased at the injured site of spinal cord after Wnt4‐modified NSCs transplantation (Figure 6A–C). PCR and WB results also showed that expression of iNOS was significantly increased in injured spinal cord after SCI. The expression of iNOS was significantly decreased and expression of Arg1 was significantly increased in injured spinal cord after Wnt4‐modified NSCs transplantation (Figure 6D–F). These results suggested that transplantation of Wnt4‐modified NSCs induced M2 polarization of macrophages to improve inflammatory micro‐environment after SCI.

**FIGURE 7:**
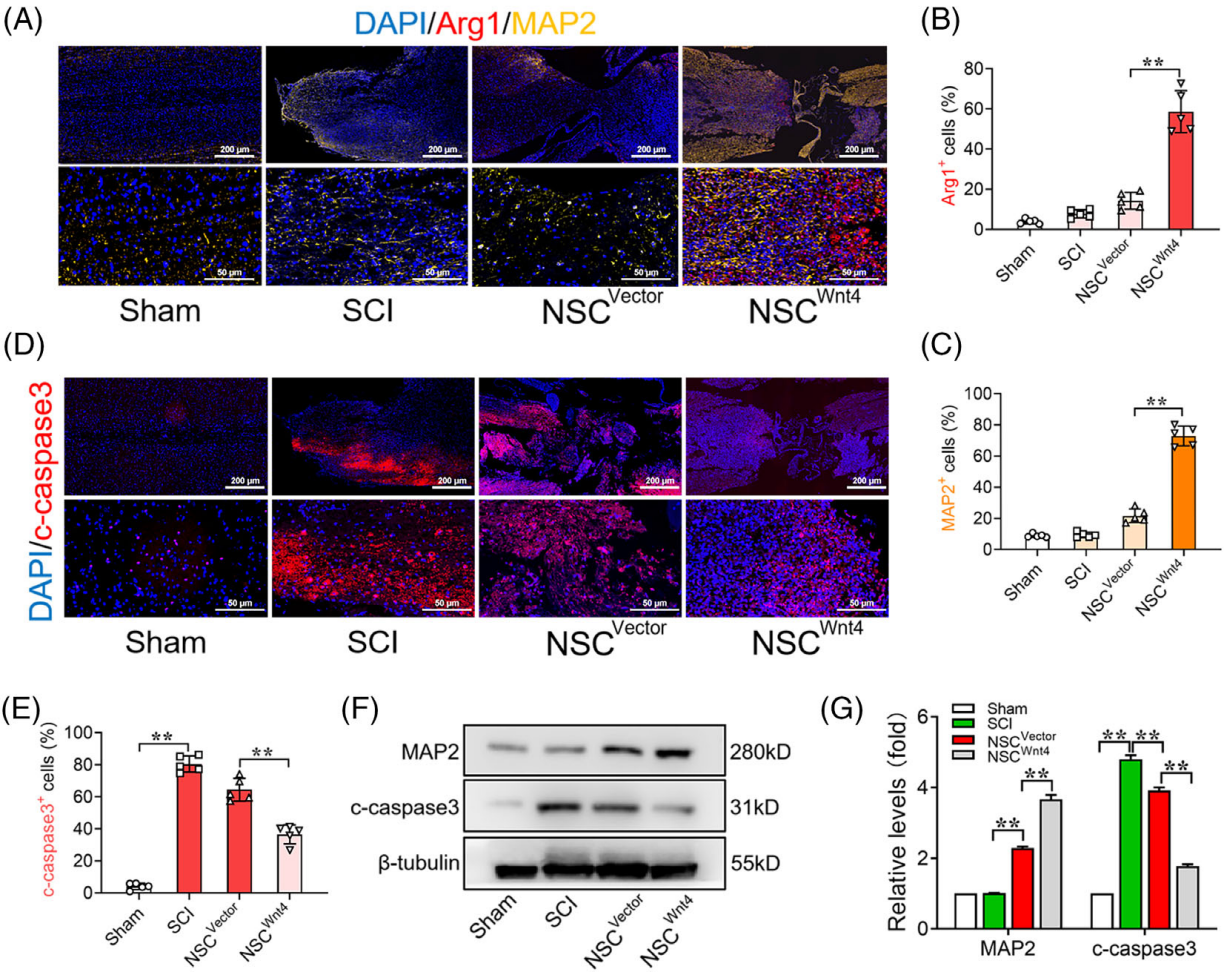
Transplantation of Wnt4‐modified NSCs improves inflammatory micro‐environment to promote axonal regeneration and suppresses tissue damaged. (A) Immunofluorescence analysis of Arg1^+^ (red) and MAP2^+^ (yellow) cells at the injured site of spinal cord in different groups, bar: 500 μm in upper row; 100 μm in lower row. (B, C) Quantification of immunofluorescence data in panel A, *n* = 5. (D) Immunofluorescence analysis of c‐caspase3^+^ (red) cells at the injured site of spinal cord in different groups, bar: 500 μm in upper row; 100 μm in lower row. (E) Quantification of immunofluorescence data in panel A, *n* = 5. (F) Western Blot analysis of expressions of MAP2 and c‐caspase3 in the spinal cord tissue in the different groups, *n* = 3. (G) Quantification of western blot data in panel F, *n* = 3. (The data are presented as the means ± SD. **p < 0.01 compared between groups; *p < 0.05 compared between groups.)

Furthermore, previous studies reported that increasing M2 cells is beneficial to axonal regeneration and tissue repair. Immunofluorescence results showed that increasing MAP2^+^ cells were along with increasing M2 cells at the injured site of spinal cord after transplantation of Wnt4‐modified NSCs (Figure 7A–C). In addition, Previous studies showed that the expression of c‐caspase3 is increased after brain injury and spinal cord injury, which further leads to the increasing of damage of the neural tissue.^42^, ^43^ The c‐caspase3^+^ cells were significantly increased at the injured site of spinal after SCI and decreased after transplantation of Wnt4‐modified NSCs (Figure 7D,E). WB analysis showed the similar results of the expressions of MAP2 and c‐caspase3 in the injured spinal cord (Figure 7F,G). These results indicated that transplantation of Wnt4‐modified NSCs improved the inflammatory micro‐environment to promote axonal regeneration and suppress tissue destruction after SCI.

### 3.7 Wnt4‐modified NSCs tended to differentiate into neuron to promote tissue repair and locomotor recovery

**FIGURE 8:**
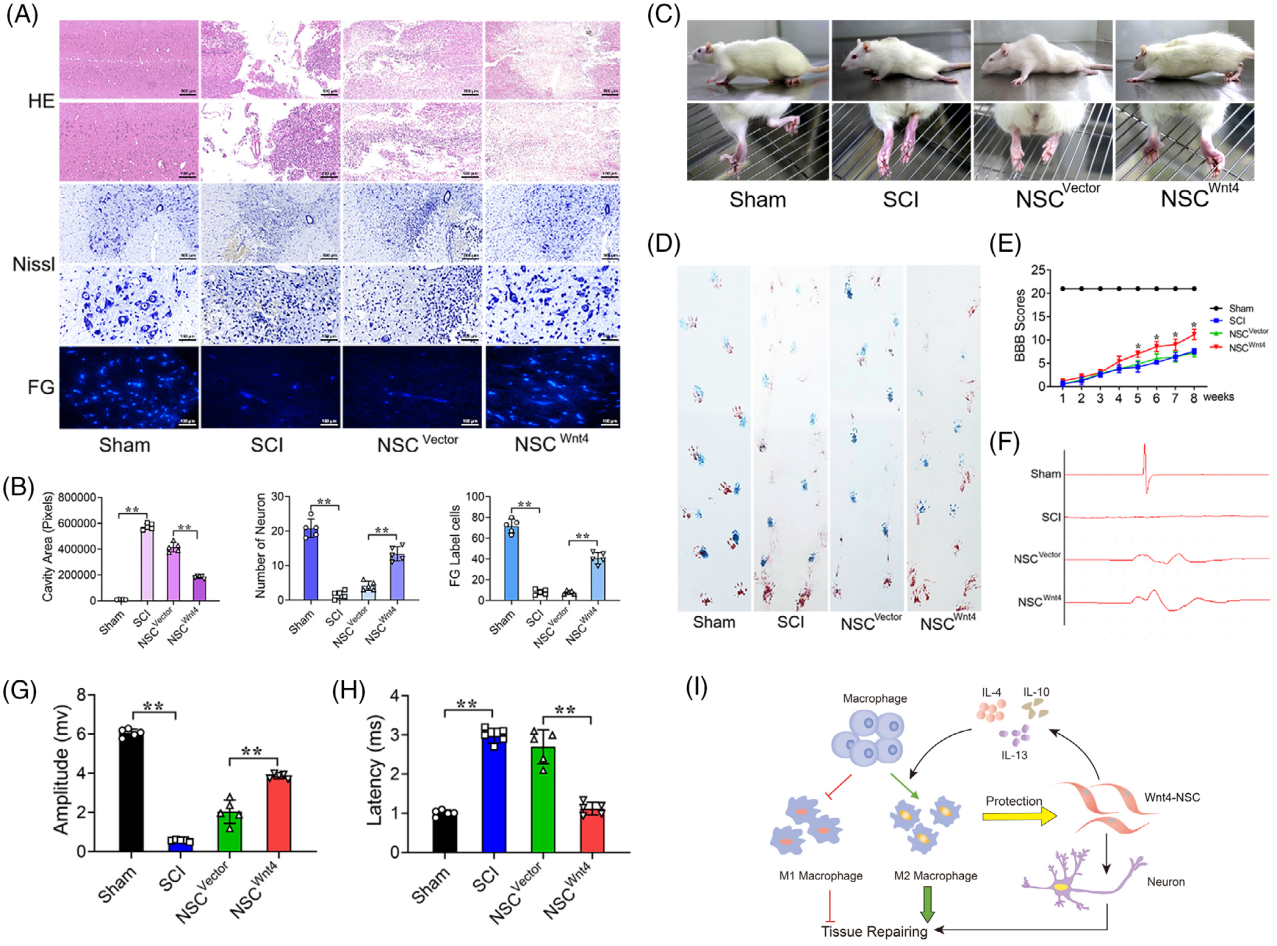
Wnt4‐modified NSCs tended to differentiate into neuron to promote tissue repair and locomotor recovery. (A) HE, Nissl and Fluorogold (FG) staining of spinal cord in different groups, bar: 500 μm in upper row; 100 μm in lower row. (B) Quantification of HE, Nissl and FG staining in different groups. (C) Behavioural character images showing hindlimb movement in different groups. (D) Footprint analysis of different groups, blue is forelimb, red is hindlimb. (E) BBB scores in different groups. (F) Electrophysiological outcomes of spinal cord evoked potential (SCEP) in different groups. (G, H) Quantification of SCEP latency and amplitude. (The data are presented as the means ± SD, *n* = 5. **p < 0.01 compared between groups.) (I) A schematic diagram of the current study: This study demonstrated that the interaction between transplanted cells (Wnt4‐modified NSCs) and macrophages in micro‐environment of SCI. Wnt4‐modified NSCs improved the inflammatory micro‐environment through increasing of M2 polarization in macrophages to facilitate axonal regeneration and tissue repair.

The appropriated micro‐environment is to the benefit of neuronal differentiation of NSCs. We further investigated the differentiated status of the drafted cells. Immunofluorescence results showed that MAP2^+^ positive NSCs (GFP^+^) were significantly increased and GFAP^+^ positive NSCs (GFP^+^) were significantly decreased at the injured site of spinal cord after transplantation of Wnt4‐modified NSCs (Figure S9A–D). Furthermore, HE, Nissl and Flurogold staining indicated that the cavity was significantly smaller and there was more survival neuron at the injured site of spinal cord after transplantation of Wnt4‐modified NSCs (Figure 8A,B). Furthermore, behavioural test including BBB motor function scores, footprint analysis and SCEP result demonstrated that transplantation of Wnt4‐modified NSCs improved the motor function of hindlimb in SCI rats (Figure 8C–H). These results indicated that Wnt4‐modified NSCs tended to differentiate into neuron to promote tissue repair and locomotor functional recovery.

## 4 DISCUSSION

Macrophages play an important role in the inflammatory response of injured spinal cord. These cells have the ability to polarize into two phenotypes: M1 and M2 cells. The ratio of these two cell subsets determines the homeostasis of the local micro‐environment.^41^ M1 cells primarily regulate innate immunity to clear foreign pathogenic microorganism and wound debris. M2 cells release various anti‐inflammatory cytokines and growth factors to regulate inflammatory responses and promote tissue repair.^18^ In general wound, M1 cells is mainly presented in the initiate stage of inflammation and switch to M2 to begin tissue repair in the latest stage. However, M1 cells persisted in the micro‐environment of SCI, resulting in irreversible damage to the spinal cord.^19^ Thus, it is significant to simultaneously achieve valid M2 ratio to promote tissue repair in the micro‐environment for the treatment of SCI.

The micro‐environment of the injured spinal cord is characterized by complex and imbalance. The imbalance micro‐environment is considered to be the principal reason of the poor regeneration and recovery of SCI.^15^ The cellular level imbalance in the micro‐environment mainly involves the differentiation of stem cells and transformation phenotypes of microglia and macrophages.^15^ After SCI, astrocytes and other neural cells can release lot of inflammatory cytokines such as IL‐1β, TNF‐α, to induce the M1 activation of macrophages that cause a sustained imbalance in the M1/M2 ratio. We reached the conclusion that macrophages and astrocytes were increased after SCI by scRNA‐seq analysis. Macrophages were the most important contributor and modulator in this inflammatory environment. In addition, astrocytes were activated and differentiated into scar‐forming status due to an increase in macrophages (Figures 1, 2, 3, S3 and S4). It is worth noting that there was increasing of activated microglia after SCI, however, the inflammatory signal pathways in microglia were suppressed which indicated that microglia may not be involved in the modulation of inflammatory response after SCI (Figure S3).

Cell transplantation has emerged as a potential strategy to promote tissue repair after SCI.^2^, ^6^ The primary mechanism of cell transplantation mediate functional improvement and tissue repair is axonal regeneration, neuroprotection and immunomodulation.^2^ Previous studies mainly focus on expression and secretion in various cytokines from different immune cells after SCI. Previous study also considered that NSPC transplantation improves hindlimb movement and it is able to increase T cells and decrease B cells.^44^ MSCs also were considered to modify the immune response by elevating of anti‐inflammatory and reducing of proinflammatory cytokines after SCI.^45^, ^46^ However, whether transplanted cells directly alter the inflammatory micro‐environment and the direct interactions between transplanted cells and immune cells remain unclear. In the current study, we provided first evidence to investigate the interactions between NSCs and macrophages using our co‐cultured system. In one hand, Wnt4‐modified NSCs could secret various anti‐inflammatory cytokines and induces M2 polarization of macrophages (Figures 4 and S5). On the other hand, M2 cells promote neuronal differentiation of NSCs through MAPK/JNK signal pathway (Figures S7 and S8).

Previous studies have indicated that M1 polarization of macrophages resulted in the activation of the NF‐κB/p65 signalling pathway.^47^, ^48^ This activation was also regulated by many other signal pathways, such as TLRs, AMPK.^49^ TLR4 was considered to play a significant role in inflammation mediated by macrophages and promote the nuclear translocation of NF‐κB, thereby inducing the expression of multiple inflammatory cytokines.^50^, ^51^ Thus, we explored that whether TLR4 and NF‐κB/p65 signal pathways were involved in the interaction between macrophages and NSCs. Our results showed that the expression of TLR4 was decreased and NF‐κB/p65 signal pathway was suppressed in macrophages co‐cultured with Wnt4‐modified NSCs. These results suggested that the suppression of M1 macrophages polarization was associated with TLR4/NF‐κB signalling pathways (Figure 5).

NSCs transplantation is considered a potential treatment for SCI because NSCs can differentiate into neurons and oligodendrocytes for neuronal rewiring and recruitment in the lesion.^1^, ^2^ However, previous studies have reported that most NSCs transplanted into lesion have differentiated into astrocytes rather than neuron due to the inflammatory micro‐environment of SCI.^6^, ^8^ Therefore, assisting agents that maintain a good rate of neuronal differentiation and improve the inflammatory micro‐environment are the research direction for the treatment of SCI. Wnt4 has been considered to have neurogenesis and anti‐inflammatory capacities as an important ligand of non‐canonical Wnt signal pathway.^22^, ^23^, ^24^, ^52^ Previous studies reported that Wnt4 suppressed p65 translocation and NF‐κB activation through transforming TAK1 in macrophages.^52^ Our previous studies reported that Wnt4 promotes neuronal differentiation by activating both Wnt/β‐catenin and MAPK/JNK signal pathways. Wnt4‐modified NSCs transplanted into the lesion efficiently differentiated into neurons and promoted functional recovery after SCI.^24^ In the current study, we also conducted a series of in vivo experiments to prove the beneficial therapeutic effect of Wnt4‐modified NSC. Our results showed that transplantation of Wnt4‐modified NSCs promoted M2 polarization and suppressed M1 polarization, which resulted in the propensity of macrophages to polarize into M2 cells at the injured site of spinal cord (Figure 6). The increased M2 cells provided an appropriate micro‐environment, which was beneficial for axonal regeneration and suppressing of cell apoptosis and tissue destruction at the injured site (Figure 7). Thus, the drafted NSCs tended to differentiate into neuron rather than astrocytes in this appropriated micro‐environment after SCI (Figure S9) and these effects may facilitate tissue repair and functional recovery after SCI (Figure 8, Video S1) due to promotion of neurite growth and synaptic plasticity.

Previous studies reported that transplantation of NSCs combined with biomaterial scaffolds provided promising tissue engineering strategy in SCI treatment, for which were potential to improve the survival rate of drafted cells and axonal generation. Yao et al.^53^ proposed the innovative and efficient technique of Magnesium‐Encapsulated Injectable Hydrogel and 3D‐Engineered Polycaprolactone Conduit, which can promote the repair and functional recovery of nerve injury, proving the potential application of biomaterials in the nervous system. Yuan et al.^54^ developed a cellular adaptive neurogenic (CaNeu) hydrogel delivery carrier. The dynamic network of CaNeu hydrogel loaded with ADSC could enhance axon growth and induce the transformation of M2 to establish an anti‐inflammatory micro‐environment, inhibit neuroinflammation and apoptosis, and promote nerve repair. Thus, it is worth to perform further research to establish Wnt4‐modified NSCs combined with biomaterial delivery carrier and further investigate therapeutic effect of this cell transplantation in SCI treatment.

## 5 CONCLUSION

In summary, our finding first provides evidence to confirm the interaction between Wnt4‐modified NSCs and macrophages and the involved downstream mechanism by using co‐cultured system. Transplantation of Wnt4‐modified NSCs effectively improves the inflammatory micro‐environment through inducing M2 polarization and suppressing M1 polarization of macrophages after SCI (Figure 8I). Considering these positive therapeutic effects, Wnt4 may also have remarkable potential assistant agent in NSCs transplantation for SCI.

## AUTHOR CONTRIBUTIONS

Baiqi Pan and Xiaoyu Wu contributed equally to this work. *Study design*: Yong Wan and Xiang Li. *Conducted the study*: Baiqi Pan, Xiaoyu Wu and Xiang Li. *Data collection*: Baiqi Pan, Xiaoyu Wu, Xiaolin Zeng, Jiewen Chen and Wenwu Zhang. *Data analysis*: Baiqi Pan, Xiaoyu Wu, Jiewen Chen, Xiaolin Zeng and Yong Wan. *Data interpretation*: Baiqi Pan, Jiewen Chen, Xiaolin Zeng and Xing Cheng. *Drafted the manuscript*: Baiqi Pan, Xiaoyu Wu and Xiang Li. *Revised the manuscript content*: Baiqi Pan and Xiang Li. *Approved the final version of the manuscript*: Xiang Li and Yong Wan. All authors take responsibility for the integrity of the data analysis.

## FUNDING INFORMATION

This work was supported by National Natural Science Foundation of China (Grant no. 81971151, 82102528) and Guangdong Natural Science Foundation, China (Grant no. 2020A1515010265, 2020A1515110679, 2021A1515010358).

## CONFLICT OF INTEREST STATEMENT

None of the authors have conflicts of interest to disclose.

## Supporting information


**Data S1.** Supporting Information

**Video S1.** The video of hindlimb locomotor function of rats in different groups.
The video showed the hindlimb locomotor function of sham rats (A), SCI rats (B), SCI rats with control NSCs transplantation (C), SCI rats with Wnt4‐modified NSCs transplantation (D).

## Data Availability

The datasets used and/or analysed during the current study are available from the corresponding author on reasonable request.
