# Supplementary material for: Transplantation of Wnt4‐modified neural stem cells mediate M2 polarization to improve inflammatory micro‐environment of spinal cord injury
Source: Cell Prolif. 2023 Feb 6;56(8):e13415. doi: 10.1111/cpr.13415 (PMC10392051; doi:10.1111/cpr.13415)
Supplement: Supplementary file 1 — Data S1. Supporting Information [file CPR-56-e13415-s001.docx]

**Supplementary material**

Other method and material

Detailed results of Figure S3

Detailed results of Figure S4

Detailed results of Figure S5

Detailed results of Figure S8

Detailed results of Figure 8

Supplementary Fig. 1. M1 and M2 polarization of macrophages.

Supplementary Fig. 2. Transduction efficiency in NSCs

Supplementary Fig. 3. Molecular profile of microglia subtype heterogeneity acutely after SCI

Supplementary Fig. 4. Molecular profile of astrocytes subtype heterogeneity acutely after SCI

Supplementary Fig. 5. Establishment of NSCs-macrophages co-cultured system

Supplementary Fig. 6. Wnt4 induce NSCs to secrete multiple M2-polarization relative cytokines

Supplementary Fig. 7. M2 cells promote NSCs differentiate into Neuron in vitro

Supplementary Fig. 8. M2 cells promote neuronal differentiation of NSCs through activation of MAPK/JNK signal pathway

Supplementary Fig. 9. Wnt4-modified NSCs tended to differentiate into neuron rather than astrocytes

Supplementary Table. 1. primers for qRT-PCR analysis of gene expression

**Other method and material**

**Transduction**

NSCs in log phase were plated at a concentration of 1x10^5^ cells/ well in 6-well plates and transduced with the control lentivirus, Wnt4-overexpressing lentivirus (LV-Wnt4), in MEM-α with 10% FBS. Polybrene at a concentration of 10 mg/ml was added as an enhancer reagent to improve transduction efficiency (Fig. S2A to C). In addition, MOI value was used as 20 for better cell growing and the higher survival rate. After 8 hours, supernatant was changed with fresh medium. Cells were harvested for injection into the spinal cord injury model after transduction, respectively.

Establishment of NSCs-macrophages co-cultured system

To investigate the interaction between NSCs and macrophages, 6.5 mm filter with a pore size of 8.0 μm transwell chambers (Corning, Acton, MA) were used to establish co-culture system of NSCs and macrophages. To investigate the effect of NSCs on polarization of macrophages, NSCs were plated at a concentration of 5000 cells/well in the upper chambers with neural differentiation medium and macrophages were plated at concentration of 5x10^4^ cells/well in lower chambers with DMEM. To investigate the effect of macrophages on neural differentiation of NSCs, M1 or M2 cells were plated at a concentration of 5000 cells/well in the upper chambers with DMEM and NSCs were plated at concentration of 5x10^4^ cells/well in lower chambers with neural differentiation medium (Fig. S5A and B). The plates were incubated at 37°C in 5% CO_2_, after which cells in the lower chambers were collected to perform other experiments.

**Immunofluorescence (IF)**

The cells (n = 3 per group) and tissue sections from rats (n = 5 per group) were fixed in 4% PFA for 30 minutes and permeabilized with 0.3% Triton X-100 for 30 minutes. Then blocking was performed using 5% normal goat serum for 1 hour. The tissue sections and the cells were incubated in the primary antibodies (Information of antibodies were detailed in section of antibody information) overnight at 4°C. After washing three times in PBS, the primary antibodies were probed with the secondary antibodies goat anti-rabbit IgG (1:500, Invitrogen, Camarillo, CA) or goat anti-mouse IgG (1:500, Invitrogen, Camarillo, CA) for 1 hour at room temperature. Finally, the coverslips were washed in PBS three times and mounted using Prolong Gold Antifade Reagent containing 4′-6-diamidino-2-phenylindole (DAPI) (Molecular Probes, Invitrogen). The marker-positive cells in each visual field were counted under a fluorescence microscope (Carl Zeiss Axio Observer Z1, Zeiss, Oberkochen, Germany).

**Real-time quantitative reverse transcription PCR (RT-qPCR)**

Tissue samples were flash-frozen in liquid nitrogen then stored at -80℃. Total RNA was extracted from cells and tissues according to the manufacturer’s protocol, and 2 µg of total DNA-free RNA was used to synthesize cDNA with the ReverTra Ace qPCR RT Kit (Toyobo, Osaka, Japan). The reactions were set up in 96-well plates using 1 µl cDNA with Thunderbird SYBR qPCR Mix (Toyobo, Osaka, Japan), to which gene-specific forward and reverse PCR primers were added. QRT-PCR was performed under the following conditions: 95°C for 10 min, followed by 40 cycles of 95°C for 10 sec and 55°C for 34 sec. These analyses were performed to detect the expression of M1, M2, Neural relative markers. β-actin was used as an internal control. Primer sequence were as Table S1.

**Western blot analysis**

Tissue cell lysis protein was extracted with T-PER tissue protein extraction reagent (Thermo Scientific). Cells were lysed in RIPA buffer. Total protein was extracted, and all the protein concentration was determined with a BCA assay. Subsequently, 20 µg of total protein was loaded onto an 10% SDS-PAGE gel, and the separated proteins were transferred by electro blotting to PVDF membranes. The membranes were blocked with 5% non-fat dry milk in TBST (50mM Tris, pH 7.6, 150mMNaCl, 0.1% Tween 20) and incubated with the primary antibody (Information of antibodies were detailed in section of antibody information) overnight at 4°C. Immunolabelling was conducted using ECL reagent (Invitrogen, Camarillo, CA).

**Flow cytometric analysis and sorting**

Single-cell suspensions were prepared and were stained with fluorochrome- conjugated antibodies. Data were collected on a BD LSRII flow cytometer (BD Biosciences, San Jose, CA) and analyzed with FlowJo software (Tree star, Ashland, OR). Data were acquired as the fraction of labeled cells within a live-cell gate set for 50000 events. For flow cytometric sorting, cells were stained with specific antibodies and isolated on a BD FACSAria cell sorter (BD Bioscience).

**Antibody information**

The antibodies used for Western blot were from following source: anti-CD206 antibody (Abcam, UK; 1:1000), anti-CD163 antibody (Abcam, UK; 1:1000), anti-CD68 antibody (Abcam, UK; 1:1000), anti-β3-tubulin antibody (Abcam, UK; 1:1000), anti-MAP2 antibody (Abcam, UK; 1:1000), anti-GFAP antibody (Abcam, UK; 1:1000), anti-TLR4 antibody (CST, MA; 1:1000), anti-iNOS antibody (CST, MA; 1:1000), anti-Arg1 antibody (CST, MA; 1:1000), anti-c-caspase3 antibody (Abcam, UK; 1:1000), anti-GAPDH antibody (Sigma-Aldrich, MO; 1:10000), anti-β-actin antibody (Sigma-Aldrich, MO; 1:10000) and anti-β-tubulin antibody (Sigma-Aldrich, MO; 1:10000).

The antibodies used for IF were from following source:anti-β3-tubulin antibody (Abcam, UK; 1:200), anti-MAP2 antibody (Abcam, UK; 1:200), anti-GFAP antibody (Abcam, UK; 1:200), anti-p65 antibody (CST, MA; 1:200), anti-iNOs antibody (CST, MA; 1:200), anti-Arg1 antibody (CST, MA; 1:200), anti-c-caspase3 antibody (Abcam, UK; 1:100).

**Detailed results of Figure S3**

Microglia are the main cell type in SCI. To determine the heterogeneity within the microglia subtypes, clustering analysis was performed on microglia and visualized on a separate UMAP (Fig. S3A, C). Homeostatic microglia and activated microglia were identified based on the expression of several annotated markers, such as P2ry12 and Lgals3 (Fig. S3B, C). Homeostatic microglia were the predominant subtypes in the uninjured spinal cord and the activated microglia subtype was increased in SCI group (Fig. S3D). Contrary to expectations, the inflammatory pathway was inhibited in activated microglia subtype or SCI group (Fig. S3E to G). These results indicated that activated microglia were increased after SCI, however, these cells may not involve in the inflammatory response.

**Detailed results of Figure S4**

Based on the expression of marker genes, astrocytes were divided into three subtypes (Fig. S4A). They were visualized on Violin and UMAP plots (Fig. S4B and C). We also selected several highly expressed genes to further distinguish the three subtypes (Fig. S4E). Changes in the proportion of cell subtypes indicated that reactive and scar-forming astrocytes subtypes were significantly increased at the injured site of spinal cord (Fig. S4D). To quantitatively track astrocytes in control and injury group, we performed trajectory analysis. The results showed the differentiation paths from naive to scar-forming astrocytes and indicated naive astrocytes would be differentiated into scar-forming astrocytes after SCI (Fig. S4F). This analysis indicated that astrocytes become activated in SCI which contribute to scar formation and is to the disadvantage of tissue repair.

**Detailed results of Figure S5**

Previous studies reported that different cytokines induced macrophages to polarize into different phenotypes. M1 cells are produced after the monocyte stimulated with tumor necrosis factor-alpha (TNF-α) or Interleukin (IL) -1 ^1, 2^; M2 cells are produced after the monocyte stimulated with IL-4 or IL-13 ^1^. We first investigated whether Wnt4 could promote NSCs to generate anti-inflammatory factors. multiple cytokines array The results of multiple cytokines array showed that the signal intensity of anti-inflammatory factors including IL-4, IL-10, IL-13, IFN-γ and NT-3 were increased in NSCs treated with Wnt4 (Fig. S5A and B). Similar results of RT-qPCR and ELISA analyses showed that IL-1, IL-4, IL-13 and TNF-ɑ expressions in NSCs at mRNA and protein levels (Fig. S5C and D). These results suggested that Wnt4 induce NSCs to produce multiple anti-inflammatory cytokines which may promote M2 polarization of macrophages.

**Detailed results of Figure S8**

MAPK/JNK is a crucial signal pathway to neuronal differentiation according to our previous study ^3^. We next investigated whether MAPK/JNK was involved in promoting neuronal differentiation of NSCs by M2 cells. NSCs was pre-treated with JNK specific inhibitor (SP600125) in the NSCs-macrophages co-cultured cell system. The immunofluorescence results showed that after suppression of MAPK/JNK signal pathway in NSCs, β3-tubulin and MAP2 positive cells were significantly decreased in NSCs co-cultured with M2 cells (Fig. S8A and B). Similar results of RT-qPCR and WB analyses showed that neurogenic markers expression including β3-tubulin and MAP2 in mRNA and protein levels (Fig. S8C to E). These results suggested that M2 cells promote neuronal differentiation through activation of MAPK/JNK signal pathway.

**Detailed results of Figure 8**

To determine the tissue repair effect of Wnt4-modified NSCs transplantation *in vivo*, the lesion cavity in HE staining was calculated at 8 weeks after surgery to detect tissue repair. After injection of Wnt4-modified NSCs into the injured spinal cord, the total lesion cavity group was significantly reduced. Ventral horn motor survival neurons at lesion epicentre were calculated using Nissl staining. There were significant increase in residual motor neurons after injection of Wnt4-modified NSCs in injured spinal cord. Furthermore, we examined whether the axonal regeneration contributed to axonal plasticity via retrograde tracing using FG. FG labeled neuron was significantly increased after injection of Wnt4-modified NSCs in injured spinal cord which indicated more neural connections were generated between the two ends of the injured spinal cord after injection of Wnt4- modified NSCs (Fig. 8A and B). These results suggested that transplantation of Wnt4-modified NSCs promote tissue repair of the injured spinal cord effectively in SCI rats.

Our research confirmed that transplantation of Wnt4-modified NSCs can enhance motor function recovery in SCI rats. Wild type NSCs and Wnt4-modified NSCs were injected into the injury site to evaluate the therapeutic effect of NSCs transplantation on functional recovery. The rats in sham group could grab and step easily using the hindlimb, whereas the rats in SCI group and wild type NSCs group barely grab and stand up (Fig 8C). As expected, the rats in Wnt4-modified NSCs group could grab mildly and step slowly using their hindlimb (Fig. 8C, Supplementary video 1). To further assess hindlimb coordination, we performed footprint analysis. The results showed the perfect coordination in the rats of sham group. In contrast, incongruous gaits and extensive drag of both hind limbs were observed in rats of SCI and wild type NSCs groups. As expected, the rats in Wnt4-modified NSCs group exhibited a similar gait to the sham group (Fig. 8D). (Fig. 8D). The BBB score results also showed that the hindlimb locomotion was zero point in each group postoperatively after the operation. Over the course of 2 months, the BBB score in Wnt4-modified NSCs group was significantly higher than those of the SCI and wild type NSCs groups (Fig. 8E). To further confirm sensory and motor functional recovery, we performed electrophysiological analysis. The SCEP in the Wnt4-modified NSCs group was significantly stronger than SCI and wild type NSCs groups. The Wnt4-modified NSCs group had a shorter latency period and significantly higher amplify than SCI and wild type NSCs groups (Fig. 8F to H). These results suggested that transplantation of Wnt4-modified NSCs promote locomotor functional recovery effectively after SCI.

**Reference**

1. Milich LM, Ryan CB, Lee JK. The origin, fate, and contribution of macrophages to spinal cord injury pathology. Acta Neuropathol, 2019;137:785-97.

2. Pineau I, Lacroix S. Proinflammatory cytokine synthesis in the injured mouse spinal cord: multiphasic expression pattern and identification of the cell types involved. J Comp Neurol, 2007;500:267-85.

3. Li X, Peng Z, Long L, Tuo Y, Wang L, Zhao X, et al. Wnt4-modified NSC transplantation promotes functional recovery after spinal cord injury. FASEB J, 2020;34:82-94.

**Supplementary Fig.1. M1 and M2 polarization of macrophages**


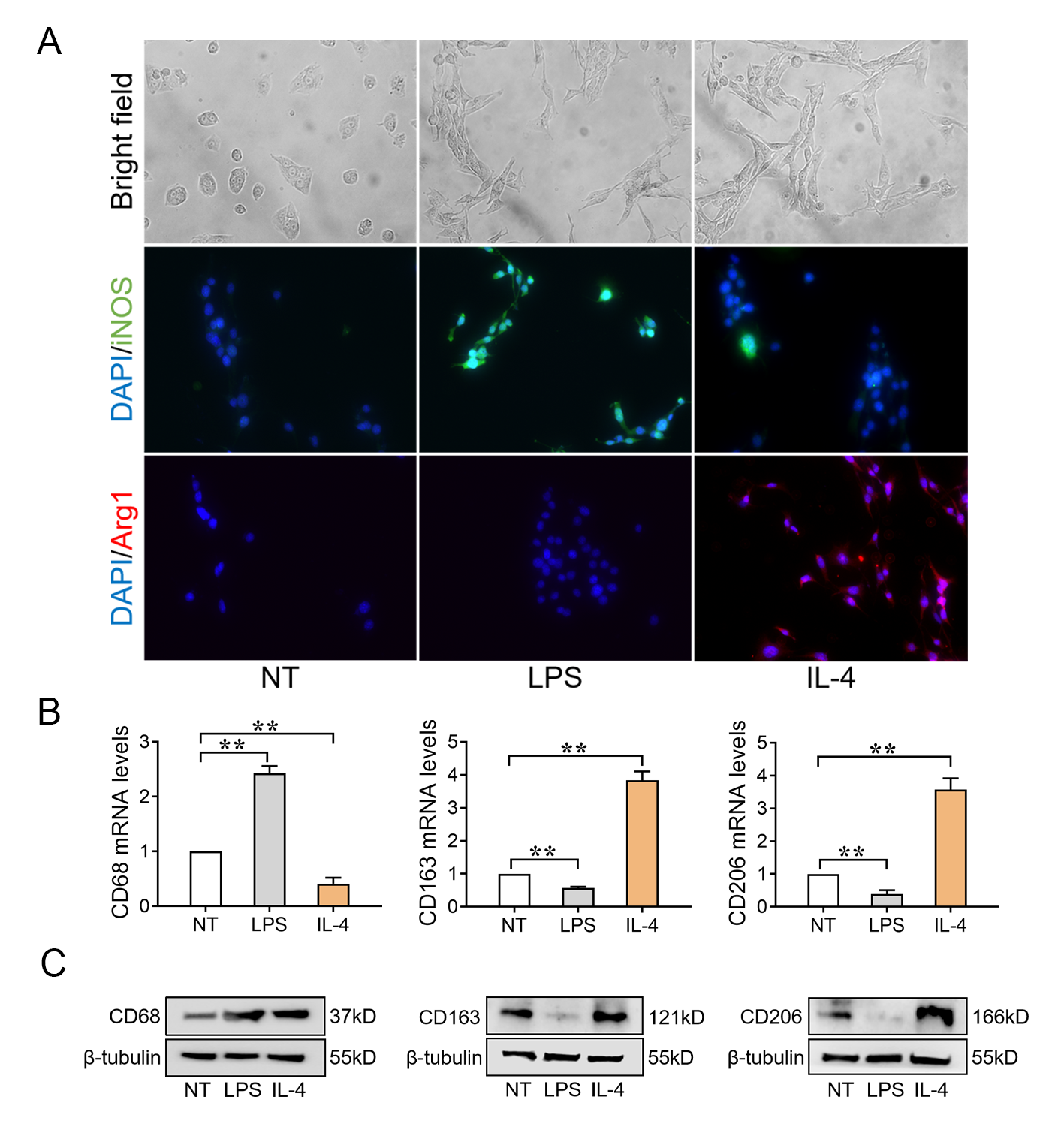


**Fig. S1. M1 and M2 polarization of macrophages.** **(A)** Bright field and Immunofluorescence staining of M1 (iNOS^+^) and M2 (Arg1^+^) cells in macrophages treated with LPS or IL-4, n=3, bar: 20μm. **(B and C)** RT-qPCR and western blot analysis of CD68, CD163 and CD206 expressions in macrophages treated with LPS or IL-4, n=3. (The data are presented as the means ± SD from one representative experiment of three independent experiments performed in triplicate. ** P < 0.01 compared between groups; * P < 0.05 compared between groups.)

**Supplementary Fig.2. Transduction efficiency in NSCs**


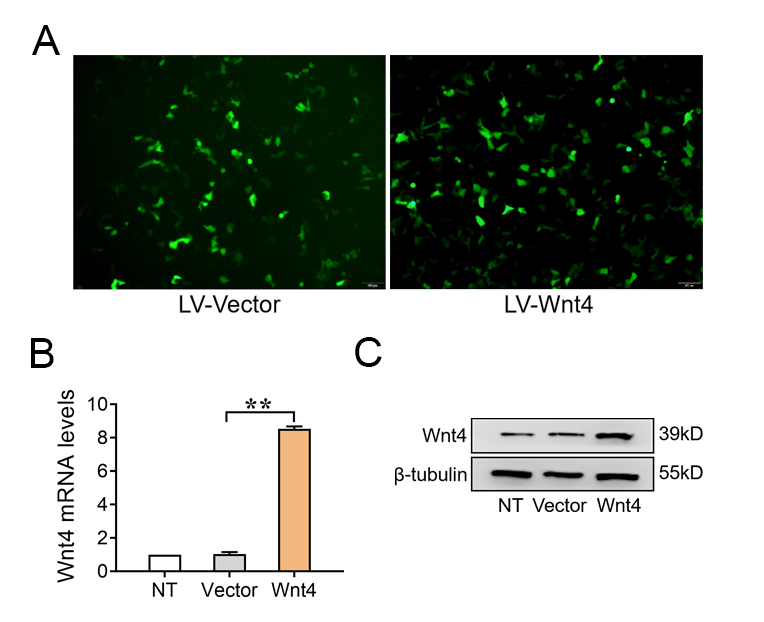


**Fig. S2. Transduction efficiency in NSCs. (A)** Immunofluorescence analysis of GFP^+^ cells in NSCs transfected with LV-Vector and LV-Wnt4, n=3, bar: 20μm. **(B and C)** RT-qPCR and western blot analysis of Wnt4 in NSCs tranfected with LV-Vector and LV-Wnt4, n=3. (The data are presented as the means ± SD from one representative experiment of three independent experiments performed in triplicate. ** P < 0.01 compared between groups; * P < 0.05 compared between groups.)

**Supplementary Fig. 3. Molecular profile of microglia subtype heterogeneity acutely after SCI**


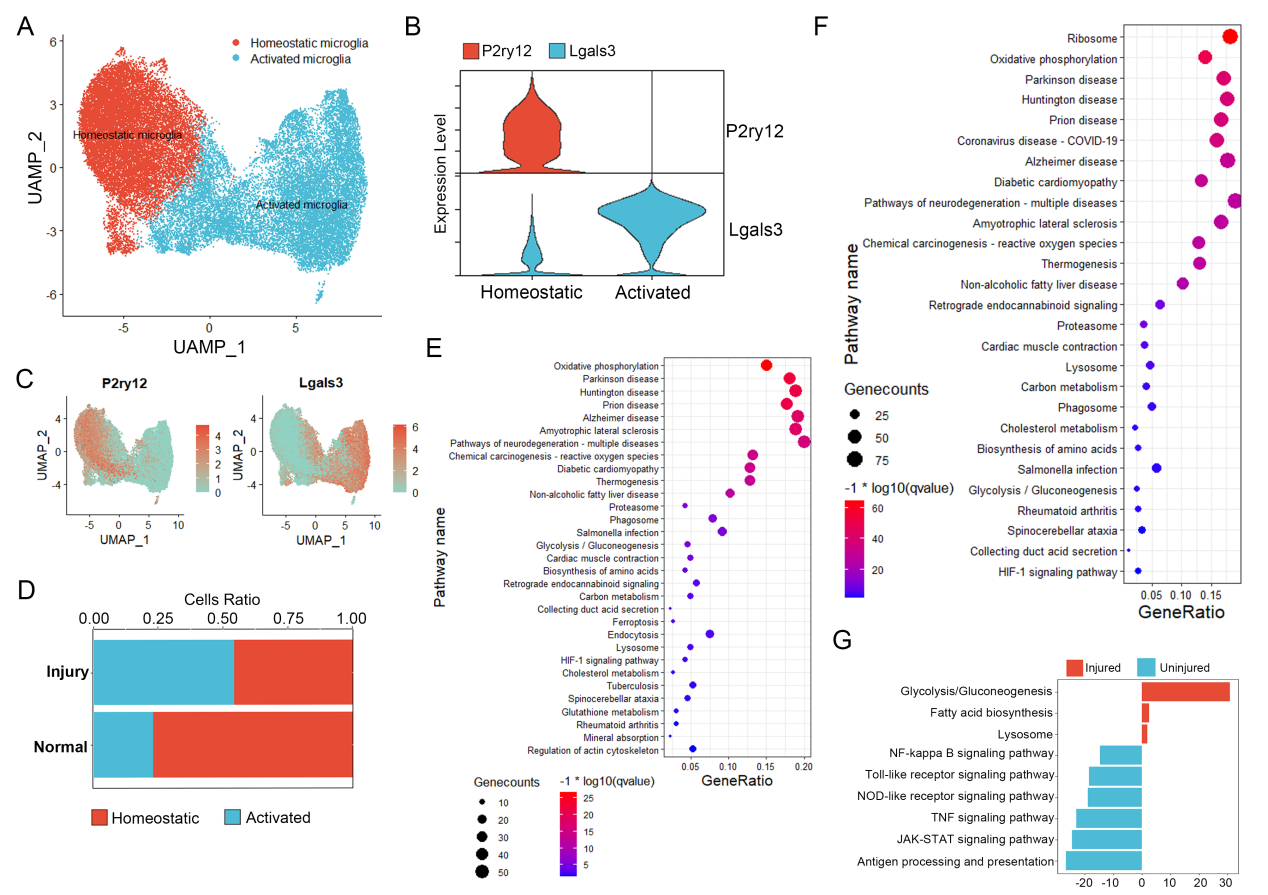


**Fig. S3. Molecular profile of microglia subtype heterogeneity acutely after SCI. (A)** UMAP plots of microglia subsets. **(B)** Violin plots showing the smoothed expression of marker genes in two microglia subsets. **(C)** UMAP plots showing the expression levels of marker genes in two microglia subsets. **(D)** The proportion of each microglia subtype in injury and control group. **(E)** KEGG Enrichment Analysis between activated and homeostatic subset. **(F)** KEGG Enrichment Analysis between injury and control group. **(G)** Gene Set Variation Analysis (GSVA) between injury and control group in microglia showing the inhibition of inflammatory pathways.

**Supplementary Fig. 4.** **Molecular profile of astrocytes subtype heterogeneity acutely after SCI**


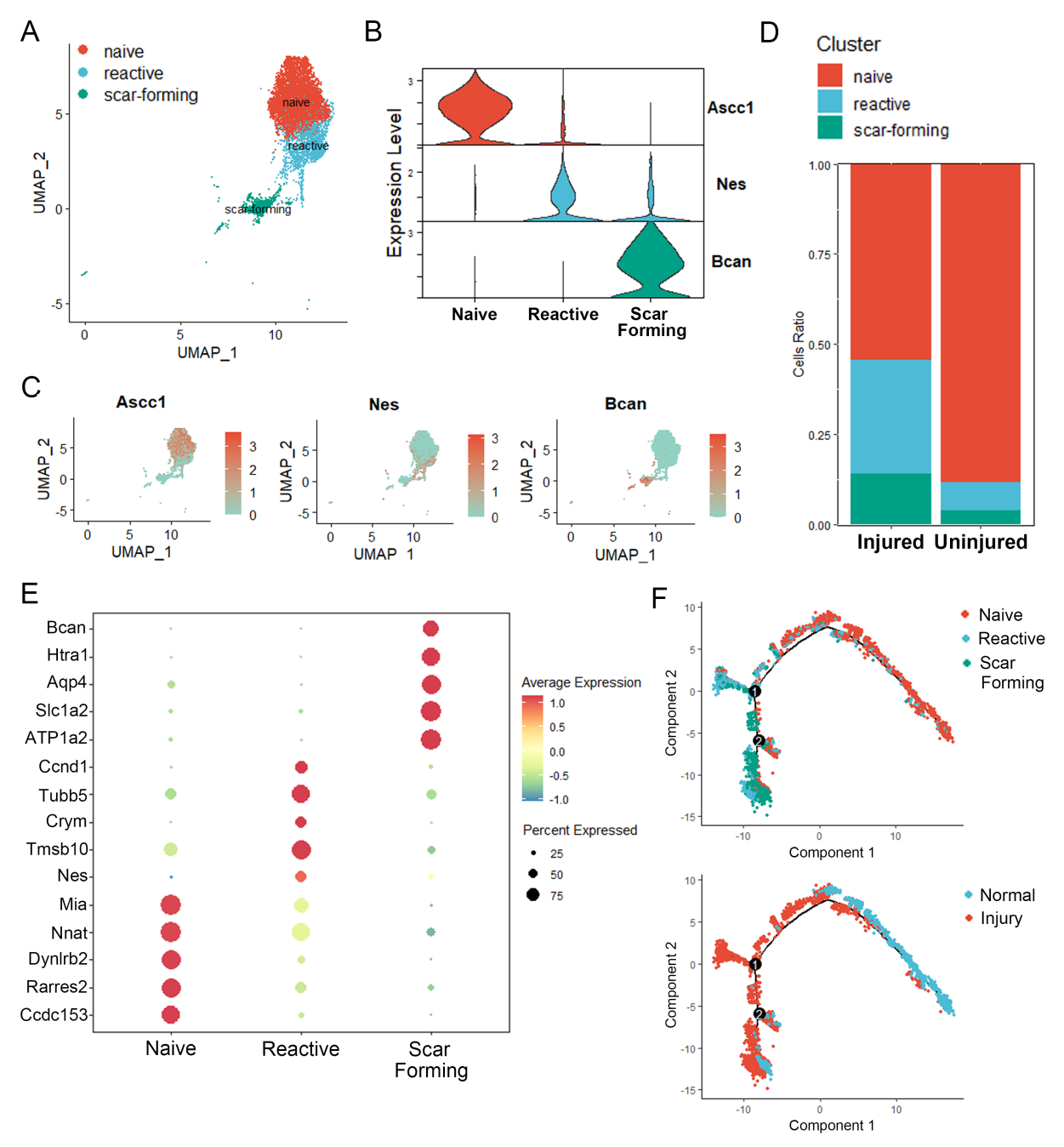


**Fig. S4. Molecular profile of astrocytes subtype heterogeneity acutely after SCI. (A)** UMAP plots of astrocytes subsets. **(B)** Violin plots showing the smoothed expression of marker genes in three astrocytes subsets. **(C)** UMAP plots showing the expression levels of marker genes for astrocytes subtypes. **(D)** The proportion of each astrocytes subtypes in injury and control group. **(E)** Dot plots showing the high expression of marker genes in three astrocytes subsets. **(F)** Monocle 2 pseudotime analysis for three astrocytes subsets (up); Monocle 2 pseudotime analysis between injury and normal group for astrocytes subsets (down).

**Supplementary Fig. 5. Wnt4 induce NSCs to secrete multiple M2-polarization relative cytokines**


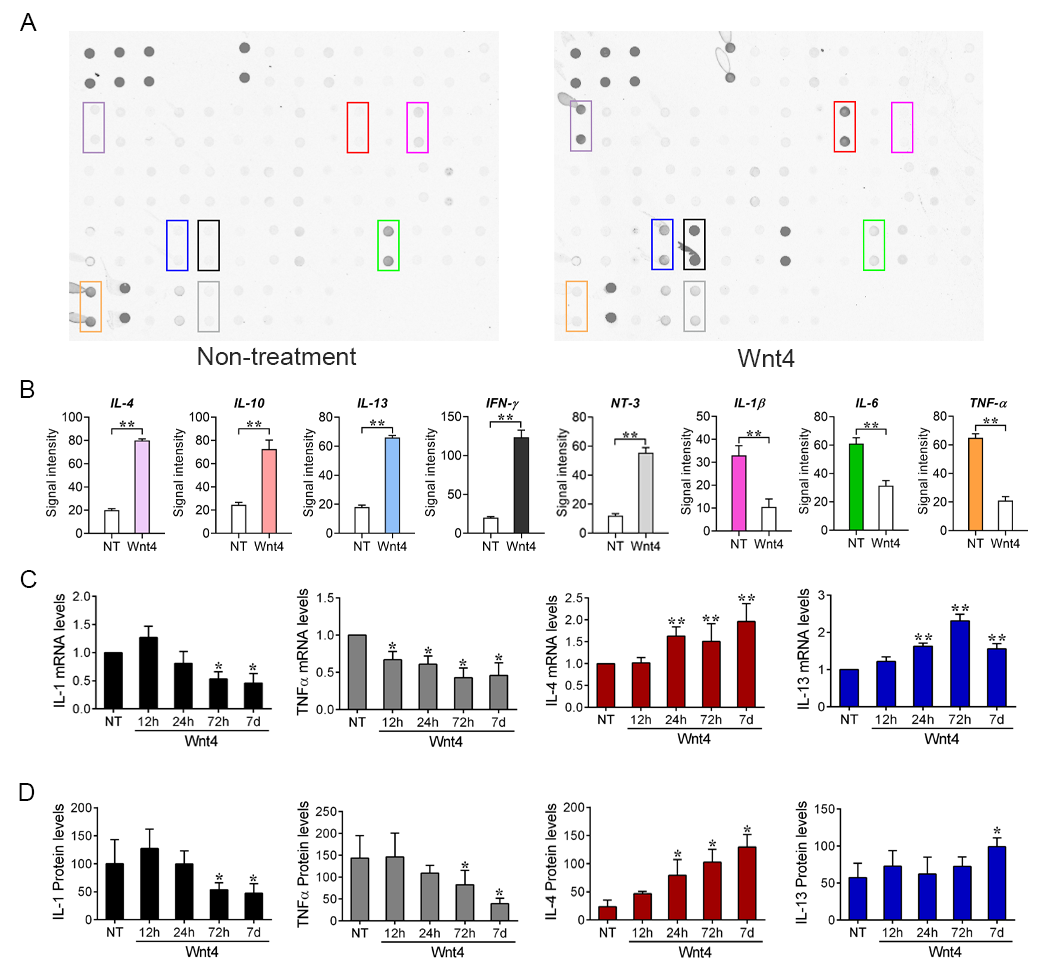


**Fig. S5. Wnt4 induce NSCs to secrete multiple M2-polarization relative cytokines. (A)** Cytokines array of NSCs treated with Wnt4. **(B)** Quantification of signal intensity data in panel A. **(C)** RT-qPCR analysis of expressions of IL-1, TNF-α, IL-4 and IL-13 in NSCs treated with Wnt4, n=3. **(D)** ELISA analysis of concentrations of IL-1, TNF-α, IL-4 and IL-13 in medium of NSCs treated with Wnt4, n=3. (The data are presented as the means ± SD from one representative experiment of three independent experiments performed in triplicate. ** P < 0.01 compared between groups; * P < 0.05 compared between groups.)

**Supplementary Fig.6. Establishment of NSCs-macrophages co-cultured system**


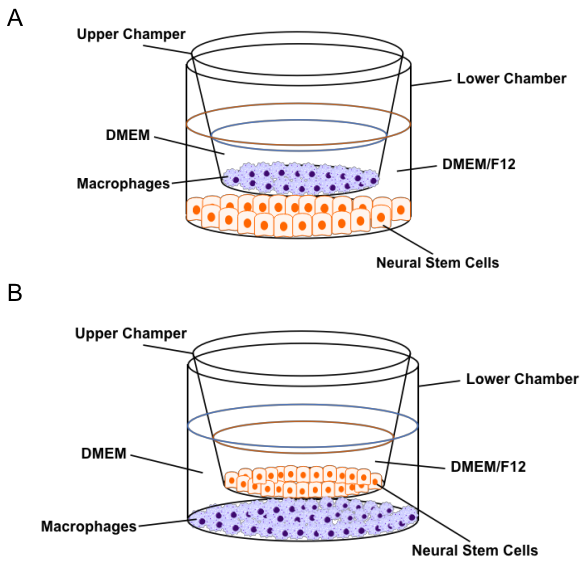


**Fig. S6. A schematic diagram of establishment of NSCs-macrophages co-cultured system. (A)** To investigate the effect of macrophages on neural differentiation of NSCs, M1 or M2 cells were plated in the upper chambers with DMEM and NSCs were plated in lower chambers with neural differentiation medium. **(B)** To investigate the effect of NSCs on polarization of macrophages, NSCs were plated in the upper chambers with neural differentiation medium and macrophages were plated in lower chambers with DMEM.

**Supplementary Fig. 7. M2 cells promote NSCs differentiate into Neuron *in vitro***


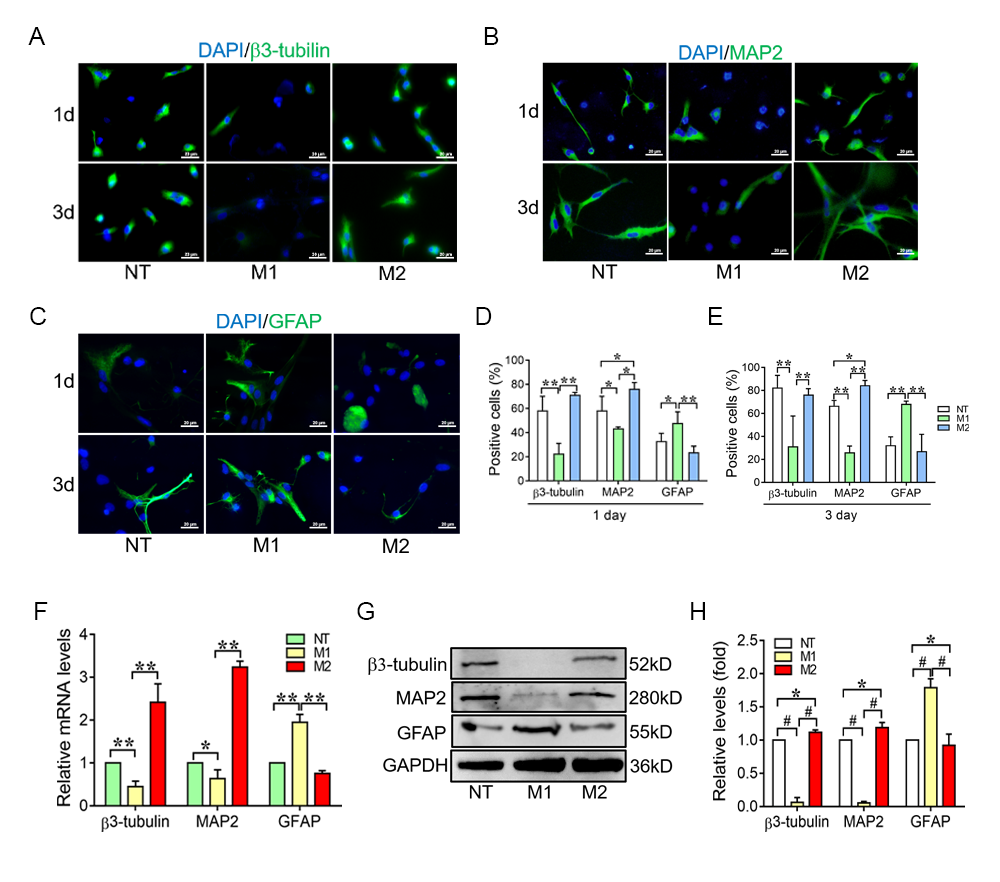


**Fig. S7. M2 cells promote NSCs differentiate into Neuron in vitro. (A)** Immunofluorescence analysis of β3-tubulin^+^ cells in NSCs co-cultured with macrophages, n=3, bar: 20μm. **(B)** Immunofluorescence analysis of MAP2^+^ cells in NSCs co-cultured with macrophages, n=3, bar: 20μm. **(C)** Immunofluorescence analysis of GFAP^+^ cells in NSCs co-cultured with macrophages, n=3, bar: 20μm. **(D)** Quantification of immunofluorescence data in panel A to C, n=3. **(E)** RT-qPCR analysis of expressions of β3-tubulin, MAP2 and GFAP in NSCs co-cultured with macrophages, n=3. **(F)** Western Blot analysis of expressions of β3-tubulin, MAP2 and GFAP in NSCs co-cultured with macrophages. **(G)** Quantification of western blot data in panel F, n=3. (The data are presented as the means ± SD from one representative experiment of three independent experiments performed in triplicate. ** P < 0.01 compared between groups; * P < 0.05 compared between groups.)

**Supplementary Fig. 8. M2 cells promote neuronal differentiation of NSCs through activation of MAPK/JNK signal pathway**


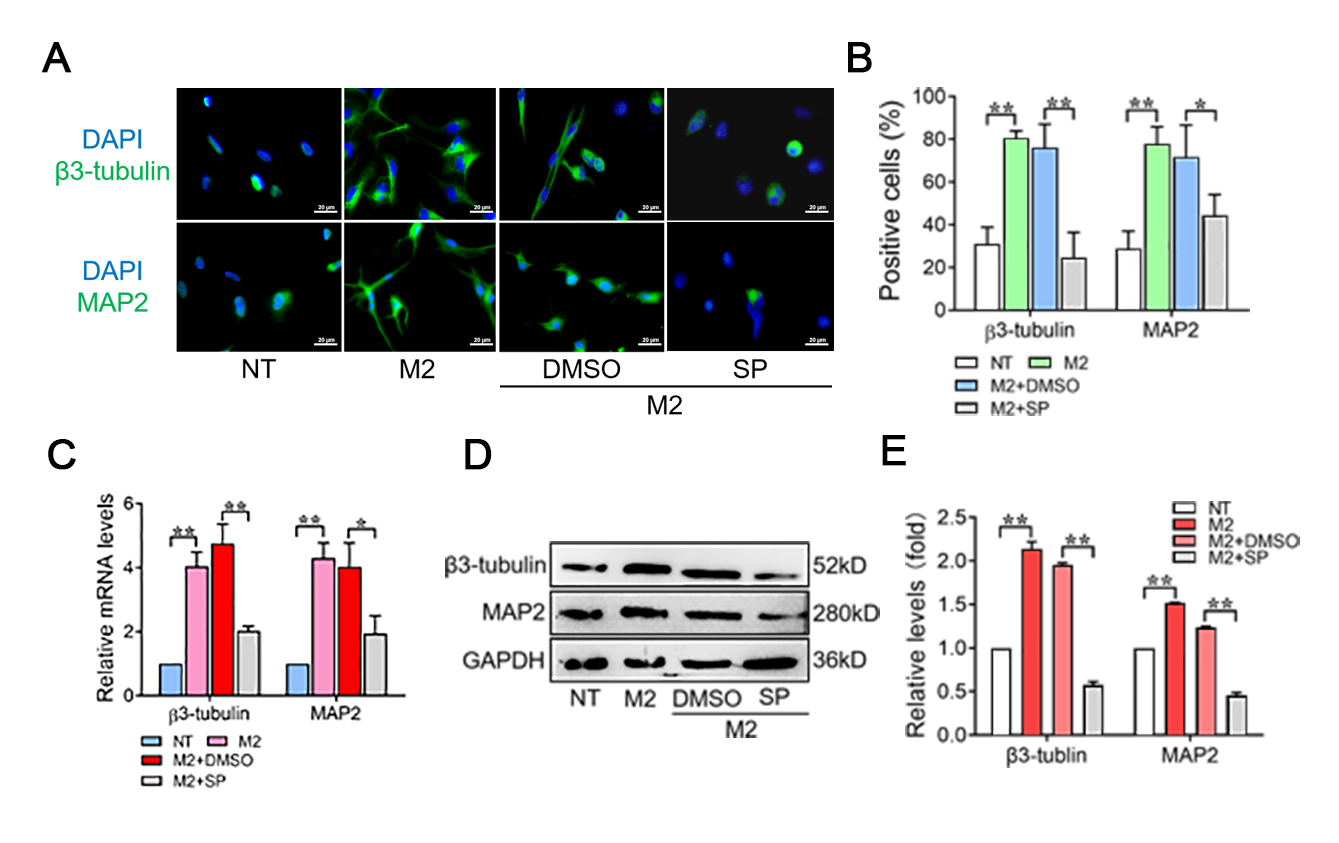


**Fig. S8. M2 cells promote neuronal differentiation of NSCs through activation of MAPK/JNK signal pathway. (A)** Immunofluorescence analysis of β3-tubulin^+^ and MAP2^+^ cells in NSCs pre-treated with JNK specific inhibitor (SP) then co-cultured with macrophages, n=3, bar: 20μm. **(B)** Quantification of immunofluorescence data in panel A, n=3. **(C)** RT-qPCR analysis of expressions of β3-tubulin and MAP2 in NSCs pre-treated with SP then co-cultured with macrophages, n=3. **(D)** Western Blot analysis of expressions of β3-tubulin and MAP2 in NSCs pre-treated with SP then co-cultured with macrophages. **(E)** Quantification of western blot data in panel D, n=3. (The data are presented as the means ± SD from one representative experiment of three independent experiments performed in triplicate. ** P < 0.01 compared between groups; * P < 0.05 compared between groups.)

**Supplementary Fig. 9. Wnt4-modified NSCs tended to differentiate into neuron rather than astrocytes**


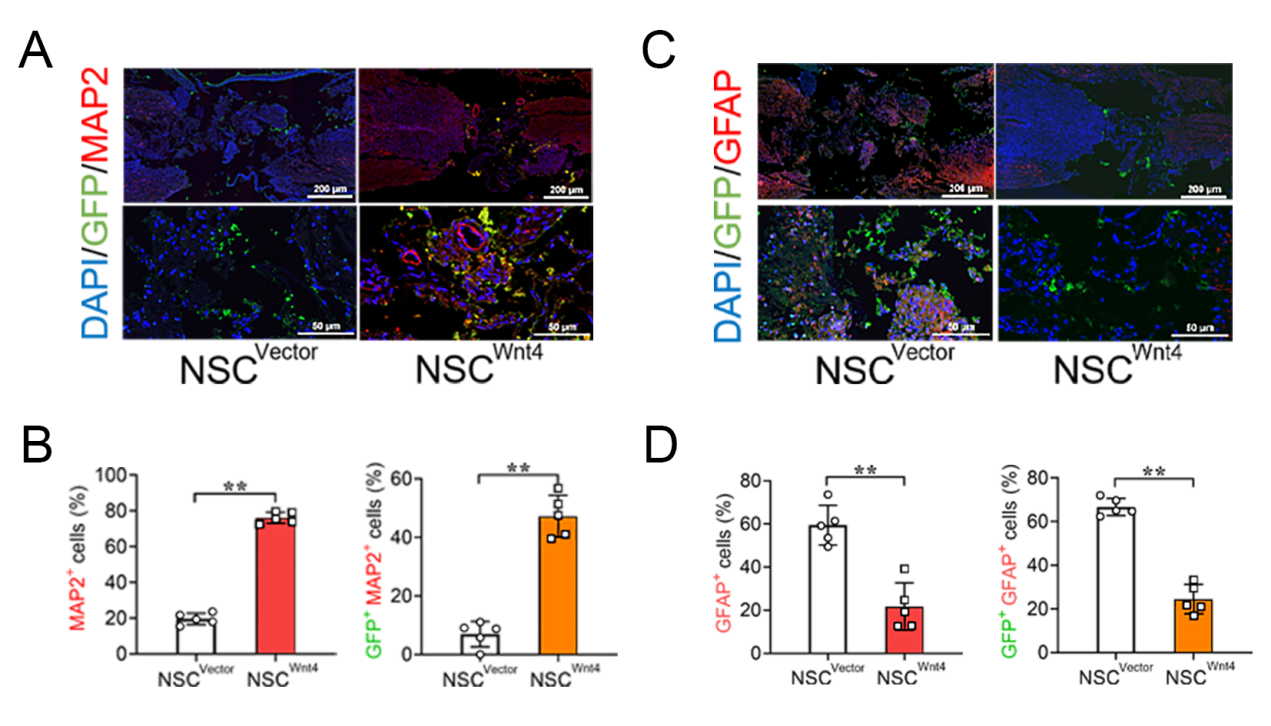


**Fig. S9. Wnt4-modified NSCs tended to differentiate into neuron rather than astrocytes. (A)** Immunofluorescence analysis of MAP2^+^ (red) and GFP^+^ (green) cells at the injured site of spinal cord in different groups, bar: 500μm in upper row; 100μm in lower row. **(B)** Quantification of immunofluorescence data in panel A, n=5. **(C)** Immunofluorescence analysis of GFAP^+^ (red) and GFP^+^ (green) cells at the injured site of spinal cord in different groups, bar: 500μm in upper row; 100μm in lower row. **(D)** Quantification of immunofluorescence data in panel C, n=5. (The data are presented as the means ± SD. ** P < 0.01 compared between groups.)

**Supplementary Table 1. Primers for qRT-PCR analysis of gene expression**

Table S1: Primer sequence for RT-qPCR

| Gene | Forward 5’-3’ | Reverse 3’-5’ |
| --- | --- | --- |
| β3-tubulin | GCAACTATGTGGGGGACTCGG | CCAGCACCACTCTGACCGAAG |
| MAP2 | GTTGGGCAGTGATTACTACGA | TTCAGGTAACTCGGACGGATG |
| GFAP | GCCCACCAAACTGGCTGAC | CTTGGACCGATACCACTCTTCT |
| CD163 | CTCAAGACACTCGGAGCCTG | CACCTGTCCGTCAGAACACA |
| CD206 | CTCTGTTCAGCTATTGGACGC | TGGCACTCCCAAACATAATTTGA |
| CD68 | TGTCTGATCTTGCTAGGACCG | GAGAGTAACGGCCTTTTTGTGA |
| IL-4 | CGTGATGTACCTCCGTGCTT | ATTCACGGTGCAGCTTCTCA |
| IL-10 | CTGGCTCAGCACTGCTATGT | GCAGTTATTGTCACCCCGGA |
| IL-13 | AACCAAAAGGCCTCGGATGT | GGCCATAGCGGAAAAGTTGC |
| TNF-α | TCTTCAAGGGACAAGGCTGC | TGGAAATTCTGAGCCCGGAG |
| IL-1 | GATCCTGCAGAGTCACGCTT | GGAATGGGCAGTGCTGTAGT |
| iNOs | CTTCGGTGCGGTCTTTTCCT | GGTGTCAGAGTCTTGTGCCT |
| TLR4 | CCTGGACTTTCAGCACTCCA | AAATCCAACACCAGGGAGGC |
| β-Actin | GCCCATCTATGAGGGTTACGC | TAATGTCACGCACGATTTCCC |
